# Identifying and selecting outcome measures for the children and families domestic abuse core outcome set

**DOI:** 10.3389/fsoc.2026.1680919

**Published:** 2026-02-18

**Authors:** Shivi Bains, Elizabeth Dunk, Jenna Harewell, Estela Capelas Barbosa, Christine Barter, Elaine Fulton, Yo Jackson, Melissa Kimber, Amanda McIntyre, Simona Skripkauskaite, Eszter Szilassy, Lazaros Gonidis, Emma Howarth, Claire Powell

**Affiliations:** 1 School of Psychology, University of Sussex, Brighton, United Kingdom; 2 Centre for Academic Primary Care, Bristol Medical School (Population Health Sciences), University of Bristol, Bristol, United Kingdom; 3 School of Health, Social Work and Sport, University of Central Lancashire, Preston, United Kingdom; 4 Elaine Fulton Consulting, Cambridge, United Kingdom; 5 Department of Psychology, Pennsylvania State University, State College, PA, United States; 6 Department of Psychiatry and Behavioural Neuroscience, Offord Centre for Child Studies, McMaster University, Hamilton, ON, Canada; 7 For Baby’s Sake Trust, Stevenage, United Kingdom; 8 Department of Experimental Psychology, University of Oxford, Oxford, United Kingdom; 9 Department of Population, Policy and Practice, Institute of Child Health, University College London, London, United Kingdom

**Keywords:** children and families, consensus, COS, domestic abuse, outcome measurement

## Abstract

**Background:**

The evidence base for child-focused domestic abuse (DA) interventions is weak. Part of the challenge is that studies measure a range of different outcomes using different outcome measurement instruments (OMI). To address this, a core outcome set (COS) comprising five outcomes was developed. The current study aimed to: (1) identify relevant OMIs and assess their quality for three outcomes in the DA-COS (*family relationships, feelings of safety, freedom to go about daily life*); and (2) reach consensus between participants on acceptable OMIs for use in research and practice contexts.

**Methods and results:**

We carried out a four-stage mixed-methods process to identify, appraise, and reach consensus on relevant tools including targeted, systematic literature searches, participant workshops to define outcome concepts, OMI appraisal of psychometrics and acceptability, and a multi-participant consensus workshop to reach consensus on OMI selection. In total, 239 OMIs were initially identified and reduced to 18 through a systematic appraisal process. Following a rating process of acceptability and feasibility, eight OMIs were taken to a final consensus workshop which resulted in the identification and provisional recommendation of two subscales from a newly developed tool for *family relationships* and *feelings of safety*. No suitable OMI was recommended for *freedom to go about daily life*.

**Discussion:**

This work is the next step toward the development of a child and family-focused DA-COS, that we hope will enable co-ordinated outcome measurement within and between practice and research. Further work is needed to adapt and evaluate the selected OMI as well as to develop a new tool to measure *freedom to go about daily life*. Work is needed to support the implementation of the DA-COS, ensure its applicability to families with diverse needs or from underserved communities and to track the benefits and potential harms of its use in this field.

## 1 Introduction

Domestic abuse (DA) is a global human rights issue with significant long-term health and wellbeing consequences for children and families (Evans et al., 2008; Oram et al., 2022; Vu et al., 2016; Walker-Descartes et al., 2021). Globally, 27% of women aged under 50 years previously or currently within a romantic relationship, are estimated to have experienced DA (Sardinha et al., 2022). In the United Kingdom (UK), 800,000 children and 450,000 adults reported experienced of DA in England and Wales in Foundations (2023). DA (also referred to as domestic violence and abuse or intimate partner violence) can involve coercive behaviors and extends beyond direct physical abuse to include economic, technological, psychological, and sexual abuse of an adult intimate partner (Sharp-Jeffs, 2021).

Children can be affected by parental DA without experiencing direct physical harm or witnessing abuse. Children exposed to DA in the home are up to four times more likely (than children who are not) to experience mental health problems such as anxiety, depression, and trauma symptoms (Doroudchi et al., 2023; Kitzmann et al., 2003). Even when clinical thresholds are not met, child survivors experience significant impairment across the lifespan from early impacts on child development during pregnancy and the first 2 years of life, to adjustment difficulties such as challenging behavior and emotional problems for young children, to negative mental and physical health outcomes in adulthood (Barlow et al., 2023; Chan and Yeung, 2009; Cunningham and Baker, 2004; Dargis and Koenigs, 2017; Holt et al., 2008; Springer et al., 2003).

Reflecting the evidence, that there is an increased risk of negative outcomes, exposure to DA is now recognized as a distinct category of child maltreatment in several settings and countries (Callaghan et al., 2018; Holden, 2003; Katz et al., 2020; Lawson, 2019; Macmillan et al., 2009). For example, in the UK, through the introduction of a landmark Domestic Abuse Bill, children are recognized as primary victims (rather than secondary or indirect victims) of DA who may need support, even in the absence of child abuse (Carlisle et al., 2024; UK Public General Acts, 2021).

Despite widespread advocacy and political will to recognize and address the impacts of DA for children and families, there is still a lack of evidence both in the UK and globally for effective interventions that reduce violence and abuse and its impacts for children and families (Allen et al., 2022; Howarth et al., 2016; Latzman et al., 2019). While there is a growing body of research in this area, systematic reviews have identified a lack of rigor in evaluation design with high levels of bias, and few replication studies (Latzman et al., 2019). In addition, the range and inconsistency in currently reported outcomes prevents the comparison of interventions and therefore reduces the possibility of synthesizing the literature (Howarth et al., 2016). Several reviews document these challenges and consequently include recommendations for consistent outcome measurement and reporting (Hameed et al., 2020; Livings et al., 2023; Weeks et al., 2024).

In the context of evaluative studies, outcome selection tends to be researcher-driven and led by availability of measurement tools, rather than children and families’ priorities for desired outcomes (Bunce et al., 2023; Howarth et al., 2015; Johnson and Stylianou, 2022; Keeley et al., 2016). Randomized controlled studies typically measure symptoms of mental ill-health or diagnoses (Howarth et al., 2016; Lorenc et al., 2020), whereas survivors, practitioners and commissioners prioritize broader outcomes focused on relationships and functioning (Howarth et al., 2015; Powell et al., 2023). Thus, outcomes selected to demonstrate the success of an intervention may not be relevant to service users or providers. This misalignment can widen the translational gap between research and practice by contributing to research wastage (Chalmers and Glasziou, 2009), mistrust in research (Smirnoff et al., 2018), and unintended consequences (Oliver et al., 2019). This has also been noted in related fields such as child mental health and neurodisability (Cohn et al., 2000; Hoagwood et al., 2012; Morris et al., 2014).

One way to encourage the use of consistent outcomes is through the creation and implementation of a core outcome set (COS) using consensus-based approaches. A COS is a minimum set of outcomes that reflect the most important outcomes by which to judge the effectiveness of a given intervention (Williamson et al., 2012, 2017). These outcomes are deemed important by all participants involved in determining the effectiveness of interventions, including those with experience of using interventions. The overall aim of using a COS is to increase consistent outcome measurement and reduce reporting bias, thereby improving the quality of evidence (Kirkham et al., 2013).

We adapted established core outcome methodology to develop a COS for evaluating child and family-focused interventions for families affected by DA (Howarth et al., 2024; Powell et al., 2025; Powell et al., 2022a). With the involvement of over 300 survivors, practitioners, and researchers, we identified five outcomes to form the domestic abuse COS (DA-COS): (1) child emotional health and wellbeing, (2) caregiver emotional health and wellbeing, (3) family relationships, (4) feelings of safety, and (5) freedom to go about daily life (Howarth et al., 2021; Powell et al., 2023, 2025; Powell et al., 2022a). The aim of the DA-COS is to provide a minimum set of outcomes that should be measured by any outcome-oriented study, with additional outcomes included as needed to capture intervention-specific impacts (Krause et al., 2021). The DA-COS was previously referred to as the Domestic Violence and Abuse Core Outcome Set (DVA-COS), however, the name was changed to align with recent UK policy changes in language (Barton-Crosby et al., 2022; Blackburn and Graca, 2021).

Despite the anticipated improvements in evidence quality, the uptake of COSs within research is generally low (Hughes et al., 2022; Williamson et al., 2022). A key barrier affecting uptake is the lack of guidance on how to measure outcomes that are identified as priorities. To address this, it is important to identify appropriate outcome measurement instruments (OMI) that can be used to assess outcomes in research and evaluation and to consider how they are implemented. Guidelines exist that detail a standardized process for selecting outcomes for COSs (Prinsen et al., 2016).

After the DA-COS development, research was commissioned to identify relevant OMIs which “mapped to” or appropriately measured the selected outcomes. Initial work to identify relevant OMIs used in DA practice found that the Warwick Edinburgh Mental Wellbeing Scale (WEMWBS) was widely used in England and Wales. Combined with its wide usage, this OMI had strong psychometric properties, and was deemed acceptable to survivors to measure two of the five outcomes included in the DA-COS, including (1) child emotional health and wellbeing, for children aged 15 years and older which is in line with previous validation research (McKay and Andretta, 2017; Ringdal et al., 2018), and (2) caregiver emotional health and wellbeing (Clark et al., 2023; Powell et al., 2022b). However, it was more challenging to identify relevant OMIs for the following three outcomes (3) family relationships, (4) feelings of safety and (5) freedom to go about daily life (Barter et al., 2026), highlighting that further work was needed.

Robust and meaningful outcome measurement is essential for evaluating the effectiveness of interventions in DA research. However, OMIs used within DA research typically lack validation and have limited evidence of psychometric properties, feasibility, and acceptability (O’Doherty et al., 2014). Additionally, OMIs within this field and adjacent maltreatment research, often measure instances of abuse rather than broader outcomes (Carlisle et al., 2024, 2025; Fallon et al., 2010; Georgieva et al., 2023; Saini et al., 2019), which reflects an earlier understanding of DA as a series of events rather than a pattern of behavior. For central constructs such as safety, these will often be operationalized as specific safety behaviors only (e.g., installing a home alarm system or making a safety plan) rather than capturing survivor perceptions of safety (Yakubovich et al., 2022). Those studies that do capture perceptions or feelings of safety develop their own study-specific tools which understandably are not validated before use (e.g., Allen et al., 2021).

While psychometric rigor, feasibility and acceptability are important when assessing the quality of OMIs, some argue that greater precedence should be given to an OMI’s acceptability over its psychometric strength (Krause et al., 2021; McCrae and Brown, 2018). Research has found that practitioners, as well as adult and child survivors of DA prefer strengths-based OMIs rather than diagnosis or deficit-focused tools which are more commonly used in research (Jacobs et al., 2024; Powell et al., 2022b; Power et al., 2024). Service users and providers report that they want OMIs to capture important contextual information for complex outcomes, such as family relationships and mental health, and that otherwise completing questionnaires can feel reductive and frustrating (Barter et al., 2026; Jacobs et al., 2024; Powell et al., 2022b; Power et al., 2024). Involving the perspectives of service users and providers can also enable researchers to understand underserved community perspectives on measurement and outcomes, which is crucial to widespread use of a COS (Femi-Ajao et al., 2020; Lowe et al., 2025; Thiara and Gill, 2010). We understand underserved communities as any community that might be considered marginalized, vulnerable, or “hard to reach.”

While the development of a DA-COS represents important progress, its utility in policy and practice remains limited without standardized, validated, and acceptable OMIs. However, few well-validated OMIs have been designed specifically for the DA field. Existing measures are often focused on diagnoses or deficits, or drawn from other disciplines, without considering their relevance or impact on children and families engaging with DA interventions.

This study aims to identify OMIs to measure the remaining three outcomes included in the DA-COS: (1) *family relationships,* (2) *feelings of safety,* and (3) *freedom to go about daily life*. This study is part of a wider program of work, including the validation of the full and short Warwick Edinburgh Mental Wellbeing Scale (WEMWBS and SWEMWBS) for a DA population for the two emotional health and wellbeing outcomes which is described separately (Harewell et al., 2026).

## 2 Methods

We carried out a four-stage OMI selection process following established approaches (Gagnier et al., 2021): (1) identifying candidate OMIs and refining the definitions of the outcomes; (2) appraising candidate OMI properties and their associated studies; (3) assessing the acceptability and feasibility of candidate OMIs; and (4) holding a consensus workshop with expert participants to select the most appropriate OMIs for each outcome. The study protocol was published with Foundations—What Works Centre for Children and Families (Howarth et al., 2025).

### 2.1 Ethics and equity considerations

We used a continuous consent model (Klykken, 2022) and ethics approval was granted by The University of Sussex’s Sciences and Technology Cross-Schools Research Ethics Committee (ER/EHH24/2, ER/EHH24/4, ER/EHH24/5, ER/EHH24/8, ER/EHH24/10). We built upon previous work involving survivors of DA (Powell et al., 2025) and had a DA adult survivor expert advisory group to support us in working in a trauma-informed way (Office for Health Improvement and Disparities, 2022). On the advice of our lived experience groups, we decided to involve young people aged 16–21 years directly and ask them to reflect back on their experiences as children, rather than involving younger participants. However, we also involved parents of younger children as well as practitioners who support younger children.

To ensure marginalized groups were involved we specifically approached service providers offering specialist services, for example to Gypsy, Roma and Traveler communities and to Black and Asian women. We aimed to ensure the workshops were accessible to participants with disabilities and additional needs through offering alternative ways to participate and pre-workshop briefings. Where we were not able to reach survivors with marginalized identities, we aimed to include service providers who worked with survivors to ensure this perspective was involved (see Supplementary material 36 for the research team’s reflexivity statement).

### 2.2 Patient and participant involvement

We had two advisory groups to give feedback on the project approach. Our survivor expert advisory group was based at VOICES Charity—a survivor-led DA organization. Our professional expert advisory group involved both practitioners and researchers with specialisms in DA, outcomes, measurement and validation. Both groups met twice over the course of the project and provided additional *ad hoc* input as needed. Members of the professional expert advisory group contributed as co-authors to this report. These advisory groups were separate from the participants described below. See Supplementary materials 1, 2 for further details about the contributions of both expert advisory groups.

### 2.3 Participants

We involved three key participant groups comprising academics, DA practitioners and survivors across the duration of this study. See Supplementary material 3 for further details on participants and 4 sample characteristics across each stage.

Academics were based in the UK and recruited through the research team’s networks and contacted directly through email. These academic participants were diverse in their specialisms, with expertise in DA, child protection research or with outcomes measurement and their OMIs.

Practitioner participants needed to be involved in DA service delivery specific to the UK. This included specialist “by and for” organizations, those providing second-tier services and those from local authority commissioning institutions. Purposeful sampling, through a key informant approach, was adopted during the recruitment process which targeted nationally regarded organizations and “by and for” services supporting minoritized survivor groups.

To ensure maximum diversity and national representation, survivor participants were recruited through five key lived-experience research networks. We recruited through pre-existing networks to prioritize survivor safety and ensure they had access to support from their respective organizations throughout the study. We recruited:

1. Young people with lived experience of DA (aged 16–21 years) who were members of Changemakers, a young people’s lived experience group affiliated with SafeLives, or of VOICES Charity. SafeLives is a leading UK DA charity, with national presence, who work alongside victims, practitioners, perpetrators, and other charities to end DA.
2. Adults over the age of 18 years, with lived experience of DA either as children or as the parents of a child under 18 years at the time of their experience. Participants were recruited through: (1) VOICES Charity, (2) Refuge’s Survivor Panel, (3) Pioneers (adult authentic voices group affiliated with SafeLives), and (4) the Domestic Abuse Commissioner’s (DAC) Survivor Platform—Voices at the DAC.

### 2.4 Process

We carried out a four-stage process to reach consensus and describe each stage in turn. Stage 1 aimed to refine our conceptual understanding of the core outcomes and identify the most relevant candidate OMIs.

#### 2.4.1 Stage 1A—rapid scoping reviews and call for evidence

Stage 1A sought to update previous research selecting OMIs for the DA-COS (Barter et al., 2026; Clark et al., 2023; Howarth et al., 2021; Powell et al., 2022a; Powell et al., 2022b). We carried out two scoping reviews of empirical studies of DA interventions and the gray literature related to current practice to capture relevant OMIs that mapped to the outcomes: *family relationships, feelings of safety* and *freedom to go about daily life* (Tricco et al., 2018). For further details on the methodology, eligibility criteria, and search strategy of both scoping reviews see Supplementary materials 5–11. In addition, a call for evidence survey was distributed between 19th August and 16th September 2024 for DA experts to provide recommendations for OMIs that might not be captured in the literature.

#### 2.4.2 Stage 1B—concept workshops

To refine our understanding of the remaining outcomes, one two-h workshop was held separately with each of the participant groups described above: practitioners (*n* = 5), academics (*n* = 4), and the Changemakers (*n* = 5). Using nominal group technique (Hall et al., 2021; McMillan et al., 2016), participants shared their interpretations of each outcome, helping to identify priority subdomains within and across groups. They discussed how candidate OMIs could best reflect these core concepts. This approach aligns with COS development principles, where early participant involvement strengthens consensus-building and the selection of appropriate OMIs (Dodd et al., 2021; Mokkink et al., 2016; Prinsen et al., 2016). Changemaker input was prioritized to ensure their insights informed subsequent sessions with practitioners and academics. Feedback was collected via Mural^1^ whiteboards to support thematic analysis by the research team.

#### 2.4.3 Stage 1C—additional searches

Additional searches were conducted to maximize the number of relevant candidate OMIs that could be mapped to the remaining three outcomes. First, to ensure the most up-to-date literature was screened for relevant publications, an additional 548 trials identified in searches of the Cochrane Library were reviewed. Second, additional searches of the non-DA literature were carried out to expand the number of candidate OMIs relevant to the remaining three outcomes. These searches were informed by the thematic synthesis of the concept workshop feedback which highlighted priority constructs comprising the core outcomes. Literature obtained through both sets of additional searches were screened by one researcher at the title and abstract and full text stages. Candidate OMIs that mapped to the core outcomes were cross-checked with previously appraised OMIs from past iterations (for further information regarding the additional searches, see Supplementary material 12, for further details regarding the studies identified across stage 1, see Supplementary material 13).

#### 2.4.4 Stage 2—appraising candidate OMIs

Once identified, candidate OMIs and their associated studies were appraised to assess their quality and scored to delineate the highest quality OMIs. This stage used the COnsensus-based Standards for the selection of health Measurement Instruments (COSMIN) and relied upon the availability of published literature to assess the quality of the candidate OMIs (Prinsen et al., 2018). Five checklists were used to assess the psychometric properties of each OMI, alongside their associated studies, and their acceptability for use within DA contexts (See Supplementary materials 14–18 for further information regarding the five checklists).

Two checklists (checklists one and two) were adapted from the COSMIN Risk of Bias (Mokkink et al., 2018) and Interpretability and Feasibility (Mokkink et al., 2016) checklists. These were used to assess various constructs of reliability and validity, including (but not limited to) content validity, internal structure and measurement properties and the clinical interpretations of the OMIs’ scoring systems. Two checklists (checklists three and four) were developed during previous work (Powell et al., 2022b). These checklists included criteria related to the psychometric properties of measures and trauma-informed factors including whether respondents are unable to answer openly and confidently due to their circumstances and whether the tool is designed in a way that prevents responders from answering freely, e.g., flagging who the administrator of a tool should be or whether the tool links the responder to other individuals/institutions. The final checklist, checklist five, assessed the acceptability of each OMI against pre-established criteria informed by interviews with a lived-experience advisory group as part of the UK Home Office-funded Children Affected by Domestic Abuse (CADA) project (Barter et al., 2026). This checklist was reviewed and approved for use by the Changemakers.

For each checklist, a scoring system was used to determine whether criteria were met (0 = unmet, 0.5 = partially met and 1 = fully met) and each OMI had three final scores:

1. A psychometric strength percentage of the total scores from checklists one to three
2. An acceptability percentage of the total scores from checklists four and five.
3. An overall total percentage, averaging the acceptability and psychometric percentages to ensure they were equally weighted.

These scores were used to rank candidate OMIs to identify the highest scoring 18 OMIs (six OMIs per outcome); these OMIs, known as the first OMI shortlist, were progressed to stage 3.

#### 2.4.5 Stage 3—assessment of acceptability and feasibility

While stage 2 appraised candidate OMIs through published evidence assessing OMI quality, stage 3 served to bolster our understanding of each OMI’s quality by assessing their acceptability and feasibility through participant feedback. One point of discussion was the acceptability of the tools to minoritized groups. Participants [Changemakers (*n* = 4), DA practitioners (*n* = 5) and academics (*n* = 7)] reviewed the research team’s appraisal of OMIs and provided their feedback during the second series of two-hour workshops. At the end of each workshop, participants voted whether to include each tool and recommend it for review in the consensus workshop. To better support the Changemakers with understanding the appraisal process and to familiarize themselves with the feedback process, a supplementary briefing workshop lasting 1 h was delivered to the Changemakers before their acceptability and feasibility workshop. Votes were summed and converted into weighted percentages, to ensure equal weighting across participant groups, and then averaged to generate an overall score. The highest scoring OMIs comprised the final shortlist of OMIs; these progressed to stage 4.

#### 2.4.6 Stage 4—the consensus workshop

A half-day multi-participant workshop was held for attendees to discuss and vote on the final shortlist of OMIs. To optimize our understanding of the final OMI shortlist, additional feedback workshops were held prior to the consensus workshop for survivors who were unable to attend the consensus workshop (because it was held in school hours) but wanted to be involved; this included the Changemakers (*n* = 4) and adult (*n* = 1) survivors. Their feedback was incorporated into the consensus workshop through pre-workshop information packs that included a copy of the OMIs, a summary of the appraisal data and commentary from the various participant groups relating to each tool (see Supplementary material 19). This was to ensure both the Changemakers’ and adult survivors’ voices were part of the consensus workshop.

**Figure 1:**
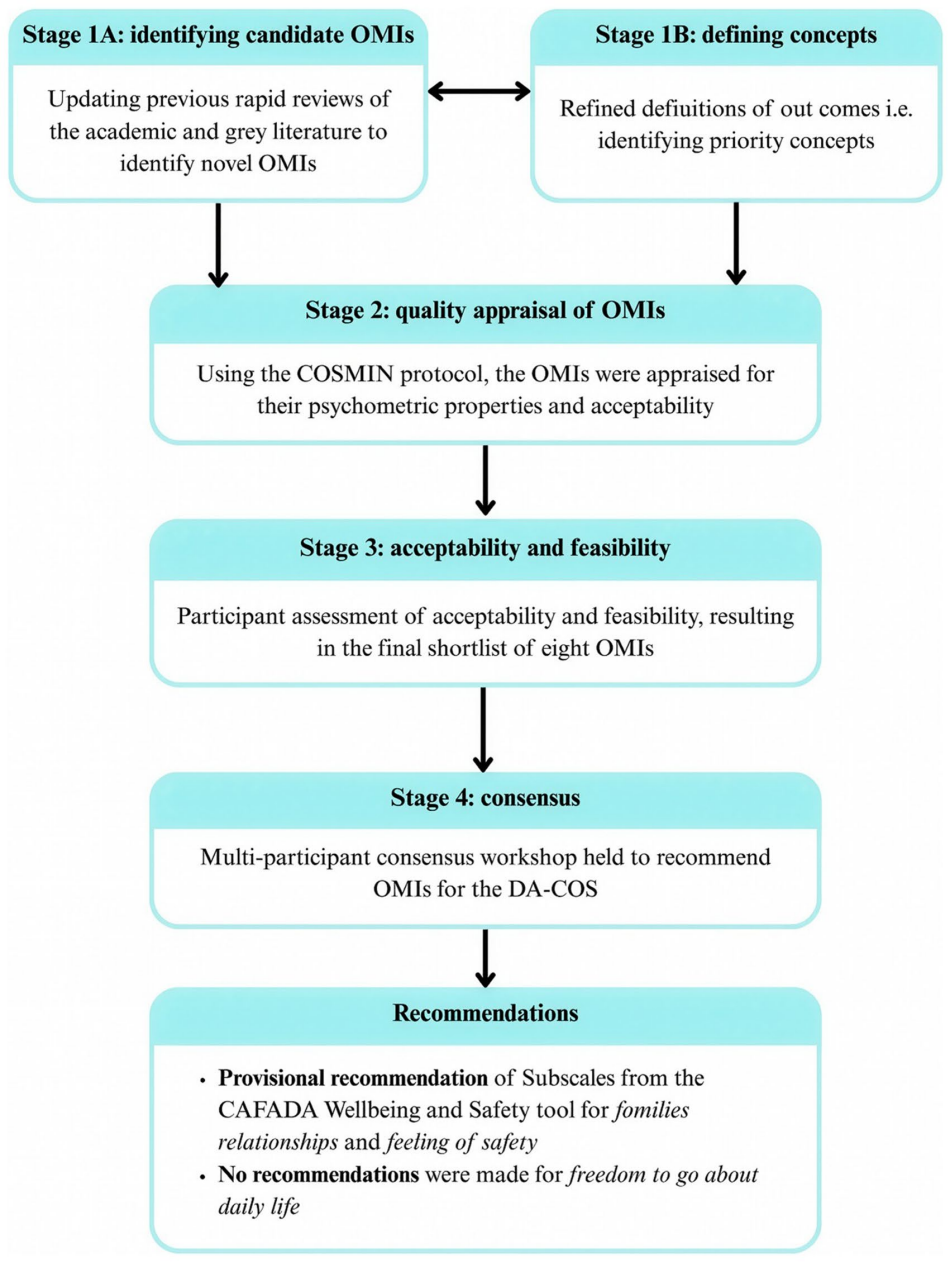
Flow diagram depiciting all the methodological stages.

A total of 29 participants attended the online workshop which included both adult (*n* = 10) and young people survivors (*n* = 1), practitioners and commissioners (*n* = 10), and academics (*n* = 8). The attendees of the consensus workshop included members of the participant groups who attended workshops in stages 1 and 3 and were supplemented by additional academics (*n* = 2), DA practitioners (*n* = 4) and survivors (*n* = 11), which included participants from the survivor expert advisory group—VOICES charity (*n* = 3). The workshop adopted the James Lind Alliance (JLA) recommendations for online consensus meetings (Jongsma et al., 2020) and learnings from past workshops (Jannesari et al., 2024; Powell et al., 2025). Participants discussed the OMIs in two rounds of small group discussions and then voted for their preferred OMI per outcome from the final shortlist. OMIs were deemed to reach consensus if they received a majority vote (over 50%) and could be selected to capture one or more of the three outcomes. In accordance with JLA consensus guidelines, participants reviewed and commented on the results. Due to software limitations, the research team were unable to analyze votes by participant group. The workshop concluded with discussions around wider considerations of the selected OMIs including possible adaptations to further include underserved populations. See Figure 1 for a diagram of the process.

## 3 Results

This study complied with the published protocol (Howarth et al., 2025). Deviations occurred, caused by restrictions due to the 2024 UK general elections, but were documented (see Supplementary material 20 for further information).

### 3.1 Stage 1—identifying candidate OMIs and refining definitions

We identified 229 OMIs from DA research and practice from our scoping reviews. After reviewing the list for duplicates from this and previous COS iterations, relevance to the core outcomes and free access, 23 candidate OMIs remained. Following our additional literature searches, based on the concept workshop findings, we identified a further 10 OMIs relevant to the core outcomes. Therefore, 33 candidate OMIs progressed to stage 2. See Table 1 for an overview of the refined definitions. For further information regarding the process of identifying, including and excluding OMIs across all stages (see Supplementary material 21). For additional information regarding the feedback from the concept workshops, the thematic synthesis of their comments and the refined definition of the outcomes: *family relationships, feelings of safety,* and *freedom to go about daily life* (see Supplementary materials 22–24).

**Table 1:** Core outcome definitions.

Core outcome	Original broad definition (Powell et al., 2022a)	Expanded definition developed from concept workshops (see Supplementary materials 22–24 for further details)	Relevant literature
Family relationships	Overall family relationships and functioning, quality and type of relationships Changes after leaving abusive partner	Includes emotional aspects such as feelings of closeness. Includes practical aspects such as conflict resolution. Need to consider relationship type.	Buchanan et al. (2014), Callaghan et al. (2016), Morrison (2015) and Stark and Hester (2018)
Feelings of safety	Global safety, including psychological, physical, body Family, neighborhood around perpetrator, at home, at school, in the community	Context-dependent: can vary by setting and by time. Includes emotional and psychological safety. Affected by relationships with others, including the parent that harms.	Barlow et al. (2023), Clarke and Wydall (2013), Goodman et al. (2014), Morrison (2024), Powell et al. (2023) and Sullivan et al. (2023)
Freedom to go about daily life	Includes ability to get home safely from school/work/friends/family, etc.	Includes practical freedom such as financial and moving/staying in locations. Includes self-freedom and choice. Includes feelings around freedom.	Herman (2015), Heywood et al. (2019), Powell et al. (2023) and Sullivan (2018)

### 3.2 Stage 2—appraising candidate OMIs

Previously identified 33 candidate OMIs were appraised at this stage to determine their quality (see Supplementary material 25 for the full list of OMIs). The majority of OMIs mapped to the outcome *family relationships* (*n* = 23), with fewer mapping to *feelings of safety* (*n* = 10) and *freedom to go about daily life* (*n* = 7). Due to the higher number of OMIs, tools mapping to *family relationships* and *feelings of safety* were assessed against a more stringent criteria, whereby OMIs were prioritized for shortlisting if they (1) had previously been used within a DA context and (2) achieved a minimum of 50% in both psychometric and acceptability scores. (These were equally weighted because of our finding from earlier work that OMIs tended to be either psychometrically valid or highly acceptable, see Clark et al., 2023). For *freedom to go about daily life*, the overall weighted percentage was used to rank the quality of the OMIs. Scores from the quality appraisal resulted in the progression of 18 OMIs, six OMIs per outcome, to stage 3.

### 3.3 Stage 3—assessment of acceptability and feasibility

Participant workshops initially aimed to review 18 OMIs (six per outcome), but the shortlist was revised following the briefing workshop with the Changemakers. For *family relationships,* one OMI—the Child and Family Against Domestic Abuse (CAFADA) “your relationships” subscale^2^—stood out for its high acceptability rating, though it lacked psychometric evaluation due to its recent development. While it narrowly missed the inclusion threshold, both expert and lived-experience groups emphasized acceptability as crucial for impact in practice. To honor the advice from both expert advisory groups, the CAFADA subscale was presented to the Changemakers to determine whether its strong acceptability could justify its use despite limited psychometric data. They strongly advocated for adding this OMI as they believed it best reflected the *family relationships* outcome. Additionally, they called for the removal of the Urban Adolescent Hope Scale (Canfield et al., 2018), which mapped to *freedom to go about daily life*, as they felt it was outdated and unrepresentative of youth experiences of hope and this outcome. To reflect lived-experience priorities, the final list comprised 18 OMIs: seven for *family relationships*, six for *feelings of safety*, and five for *freedom to go about daily life*. See Supplementary materials 26, 27 for the revised included and excluded measurement lists and their justifications.

Following discussions of the shortlisted OMIs in the acceptability and feasibility workshops, participants voted to determine the final shortlist of OMIs to be discussed in the consensus workshop (stage 4). The final vote resulted in the selection of eight OMIs (three OMIs for *family relationships*, three OMIs for *feelings of safety,* and two OMIs for *freedom to go about daily life*; see Supplementary material 28 for a description of the final OMI shortlist and Supplementary materials 29–32 for the workshop feedback on included and excluded tools).

### 3.4 Stage 4—the consensus workshop

The CAFADA wellbeing and safety scale was the highest voted OMI to capture two of the three outcomes: *family relationships* and *feelings of safety.* Participants preferred this OMI due to its high acceptability, which was attributed to the co-production of the tool with adult and youth survivors (see Supplementary material 33 for the survivor feedback workshops and Supplementary material 34 for the comments of the provisionally recommended OMIs from the consensus workshop). Due to the tool’s recent development and absence of established psychometric validation, our recommendation was limited to provisional inclusion as an OMI for the DA-COS—conditional upon further refinement and evaluation, including psychometric validation, prior to its use. No agreement was reached and therefore no recommendations were made for OMIs mapping to *freedom to go about daily life*. Table 2 provides a breakdown of participants’ votes.

**Table 2:** Consensus vote results for each OMI.

Core outcome	OMI 1	% vote for OMI 1	OMI 2	% vote for OMI 2	OMI 3	% vote for OMI 3	% votes for no OMI
Family relationships	CAFADA wellbeing and safety—relationships subscale	81.5	Medical outcomes study—social support survey	7.4	Space for action—communities and friends and family subscale	0	11.1
Feelings of Safety	CAFADA wellbeing and safety—feeling supported subscale	74.1	Roadmap (UCLAN)—your safety subscale	14.8	WHOQOL-100—safety subscale	0	11.1
Freedom to go about daily life	Space for action—subscale help seeking, competence and finances subscales	48.2	State optimism measure	14.8	N/A		

Recommended developments for the CAFADA tool were discussed. They included the use of gender-neutral language when referencing parents and the need for different versions of the measure to account for service users of different ages and cognitive maturity. Furthermore, participants highlighted the importance of testing the tool with groups from diverse and underserved backgrounds, with whole-family interventions, in services that intervene with the partner/co-parent who harms and in services operating at times of high child safeguarding risks, including when parents are especially fearful that their children may be removed from their care. On the topic of safety, participants emphasized the need to include perceptions of online safety, given the rise in technology-facilitated DA. In addition, the need for robust, trauma-informed guidance accompanying the DA-COS was underlined (see Supplementary material 35 for further details).

## 4 Discussion

This study sought to identify, appraise, and select OMIs to capture three outcomes of the DA-COS: *family relationships, feelings of safety,* and *freedom to go about daily life*. Using a four-stage process, the subscales “relationships” and “feeling supported” from the CAFADA Wellbeing and Safety Tool^3^ were provisionally recommended to capture the outcomes of *family relationships* and *feelings of safety,* respectively. This provisional recommendation reflects the need for development and validation work prior to its use in alignment with the DA-COS. Such work may conclude with refinements that would achieve applicability across the widest possible cohorts, or it may identify where particular contexts may constrain its applicability. No OMIs were selected as appropriate for capturing *freedom to go about daily life*. Overall, we identified only a few measures that had been specifically developed for use in a DA practice context and that had been psychometrically validated.

### 4.1 The CAFADA wellbeing and safety OMI

The CAFADA Wellbeing and Safety measure is a self-report tool that evaluates feelings of wellbeing and safety during a survivor’s recovery from DA. The measure comprises adult and child report (age 7 years and older) versions which include three sub-scales exploring relationships, support, and wellbeing.^4^ CAFADA subscales were identified as priority constructs within a wider project evaluating therapeutic interventions for children and families, when it became clear that no pre-existing and validated tool suitably captured these outcomes for intervention evaluation (Morrison, 2024). The CAFADA measure, co-developed with two survivor groups, is intended for use in both research and practice. Participants and advisory groups in our study agreed that the high degree of acceptability should be prioritized over psychometric assessments, which can be assessed later (Krause et al., 2021; Prinsen et al., 2016).

### 4.2 Suggested development of the CAFADA OMI

Consensus workshop participants highlighted three areas for future development of the CAFADA measure: (1) cultural and accessibility adaptations for under-served groups; (2) considerations for specific interventions; (3) psychometric evaluation of the adapted measure.

#### 4.2.1 Cultural and accessibility adaptations for underserved groups

Participants consistently identified the need to improve the tool’s cultural sensitivity and accessibility for underserved groups, across both adult and child versions. They raised concerns about its heteronormative and gendered language when referring to the non-abusive parent and the person who causes harm—highlighting that this excludes situations where the parent who harms is the mother or where families do not follow traditional structures. To address this, participants recommended using gender-neutral language and, particularly in the “relationships” subscale, allowing service users to describe their own family arrangements.

Participants suggested developing two versions of the child’s OMI to reflect developmental differences between younger children and older children and adolescents. This would also benefit some neurodivergent children who might need a visually and linguistically simpler version. Creative co-design methods could be used for ethical involvement of younger children. Additional adaptations were suggested, such as incorporating more culturally inclusive references. Given the diverse and complex needs of different populations, participants questioned whether a single universal OMI is appropriate or if creating multiple versions tailored to specific demographics would better serve those most in need. Psychometric testing would be needed in diverse and minoritized groups to ensure the tool was appropriately designed.

#### 4.2.2 Considerations for specific interventions

Participants questioned whether the CAFADA OMI was suitable for capturing the perspectives of the person who harms, for example, in whole family interventions that include both parents. The complex support required from such specialist interventions might mean this tool is not entirely suitable for use within these services (Shepherd et al., 2022). Survivor and professional participants in the study highlighted complexities for services operating where child safeguarding risks are high, especially perinatally. Parents may fear that disclosing their safety concerns and details about family relationships in an outcome questionnaire may increase the risk of their babies or children being removed from their care. This requires further exploration and potentially adaptation of the CAFADA OMI. Specialist interventions will of course need to collect intervention-specific outcomes, in addition to the core outcomes, to capture these nuances.

#### 4.2.3 Psychometric evaluation of adapted OMIs

In addition to adapting the CAFADA Wellbeing and Safety tool, further work is needed to assess the psychometric robustness of this measure to ensure it appropriately captures the constructs of interest. For inclusion in a COS, the minimum level of psychometric assessment is content validity (Prinsen et al., 2016). However, for completeness, assessments of additional reliability (scale and test–retest), validity constructs (e.g., construct and criterion), responsiveness, and measurement invariance are necessary. These assessments would ensure the CAFADA scale produced consistent and stable scores across diverse populations and captured meaningful change over time, which is especially pertinent for intervention evaluations. Alongside this, rigorous consultation processes including think-aloud exercises should be embedded into validation work to provide a holistic assessment of quality; this process should rely upon the appraisal of key participants.

#### 4.2.4 Freedom to go about daily life

The results of this consensus process failed to identify a relevant measure to capture *freedom to go about daily life*. As a survivor-driven outcome, with limited research exploring this construct, the lack of OMIs capturing this outcome was expected (Woodlock et al., 2025). However, the study improved our understanding of this outcome and how it can be operationalized for the DA-COS. During the concept workshops, participants discussed the similarities of this outcome with *feelings of safety*, and some postulated these outcomes reflect temporal differences (short- and long-term) of one “perceptions of safety” construct. Further work is needed to better understand this construct and its operationalization prior to the development and validation of a novel OMI. This work should be heavily informed by survivor perspectives. To address this, an Economic and Social Research Council-funded PhD will commence in 2025.

### 4.3 Future work

Any undertaking of the suggested adaptations should draw on systematic methodologies and continued consultation with participants. To maximize the applicability of the OMI, we recommend the involvement of a diverse participant population, comprised of survivors, practitioners, and academics. Engaging with diverse survivor populations, particularly adults and children of various ages (including during pregnancy and babies aged 0–2), those from minority ethnic backgrounds, and those with additional needs, will ensure the cultural inclusivity and accessibility of the measurement tool. Practitioners spanning a diverse range of interventions should also be involved to ensure that real-world measurement needs and challenges are addressed. This process should be repeated to capture the dynamic landscape of DA interventions and to ensure the DA-COS, as with all other COSs, adapts with changes in care over time (Maxwell et al., 2021).

To establish validity across a variety of populations, future validation work requires a sufficiently large sample size to examine measurement equivalence by relevant factors, such as culture, ethnicity, gender, intersectional needs, and the child and adult safeguarding risk levels at the time of the intervention among others. Implementing a comprehensive validation methodology of this magnitude will call for significant resources and coordination with general and specialty organizations supporting children and their families with DA. Importantly, such large samples, which are often recruited through direct liaison with services, offer the opportunity to consider the practical support required to assist services to embed high quality outcome-focused routine data collection systems into practice. The lack of high-quality routine data capture on outcomes within resource-stretched services presents a significant challenge to building the evidence base in this field (Bunce et al., 2024).

### 4.4 Implementation of the DA-COS

Limited research exists exploring the use of COSs within trials and systematic reviews and evidence that does exist indicates fairly poor uptake (Williamson et al., 2022). In these instances, a COS may contribute to the waste of research, rather than its intended reduction (Howarth et al., 2024). Further action is needed to facilitate the implementation of the DA-COS across research and practice and participants recommended the development of trauma-informed guidelines to support this. Different participants held different opinions around the purpose of the guidelines with the expert advisory group suggesting the guidelines should encourage practitioners to use a “care-first” approach when implementing the DA-COS. Commissioners outlined that guidelines should support practitioners with the interpretation of data. While the aim of collecting data typically serves to support service evaluation, which is essential to improve the quality of care, survivors raised concerns about data collection itself, questioning how the data would be used, by whom and with what purpose in mind.

Advancing measurement-based care (MBC), where practitioners use standardized OMIs for collecting routine outcome data to inform decisions about care, is important when considering trauma-informed care (Whitmyre et al., 2024). Achieved through effective collaboration with services and service users, MBC affirms care-first principles by enabling relevant, timely, and person-centered support (Wrenn and Fortney, 2017). Emerging mental health research, including studies with children and young people, indicates advancing MBC is not only possible but beneficial for trauma-informed care, however, more is needed to better understand the intricacies of advancing MBC for this population (Purbeck et al., 2020; Whitmyre et al., 2024; Zhu et al., 2021).

Future work should consider the challenges around gathering data within health and care settings, particularly when supporting those experiencing trauma and fear for their safety (Bunce et al., 2024). A range of pressures, such as under-resourcing and under-funding, make it difficult for services to prioritize the collection of robust and reliable data (Domestic Abuse Commissioner, 2024). While the implementation of a COS cannot entirely mitigate these challenges, future work exploring DA-COS implementation should consider how it can be integrated into existing systems and processes to reduce the burden on practitioners and services. This involves studying implementation across a range of service contexts—statutory and voluntary—as well as considering how the DA-COS can be adapted for use internationally.

### 4.5 Unintended consequences

There is a dearth of evidence evaluating the barriers and limitations of COSs, especially when considering the possibility of unintended consequences of implementing a COS (Howarth et al., 2024). Unintended consequences, as demonstrated through the analysis of public health policies, can arise when selected outcomes align with political priorities rather than service aims and the needs of those accessing services (Oliver et al., 2019). Implementing COSs can lessen the impact of unplanned or unrelated external influences (positive or negative “spillover” effects) which are diffuse and hard to measure.

The standardization of measurement can lead to underrepresentation of outcomes that are specific to minoritized groups within research. This, in turn, results in the commissioning of interventions that may not be suitable for minoritized groups and disadvantages smaller “by and for” organizations (NHS England, 2023; National Institute for Health and Care Research, 2020; O’Shea et al., 2017; Witham et al., 2020). Participants raised concerns that the wider use of the DA-COS might stymie innovation, for example, there would be less incentive to develop new approaches relying on alternative outcomes. This is especially pertinent when services have to compete for limiting funding and are incentivized to focus on activities and pre-specified outcomes, rather than prioritizing service users’ specific needs (Carlisle et al., 2025).

Since the development of the DA-COS, researchers have attempted to address these concerns. First, the DA-COS was developed with only a small number of outcomes which allows services to capture additional outcomes relevant to their service users’ needs without overburdening services. Throughout both the DA-COS development and the OMI selection, the research team intentionally recruited participants to ensure underserved groups were represented at all stages. However, the relevance, applicability, and *impact* of the DA-COS needs to be evaluated. This is especially relevant for determining whether the DA-COS improves service-related access and impacts for underserved groups. Commissioners and funders of service delivery can play an important role in mitigating these risks by encouraging and recommending the use of OMIs identified through the DA-COS initiative in contexts where they are appropriate, rather than mandating them for all services. At the same time, they can actively encourage evidence-based delivery and require service providers in other contexts to demonstrate their outcomes through alternative, context-sensitive means.

### 4.6 Study limitations

During the consensus workshop we were unable to track the votes based on participant group to determine any trends, this would have strengthened the transparency of the voting. Despite this limitation, we were able to reach agreement during the consensus workshop, not only through the clear majority votes for the CAFADA OMI, but facilitators of the small group discussions did not observe any differences between participant groups.

The challenge of applying the COSMIN process to the DA field has in part been the reliance on psychometric evidence when many tools are not validated. However, we hope we have addressed this through the equal weighting of the psychometric score with the acceptability score, thus taking a pragmatic approach to find the best possibility at this time.

We were unable to include as many youth survivors as we hoped during the final consensus workshop because we held the workshop during school hours. However, we held an additional youth survivor feedback workshop to capture their perspective and included their views in the workshop packs. Future work could aim to hold mixed adult/youth workshops; however, this needs further thought around facilitating equitable power sharing. A longer time period and more resources would have enabled us to work directly with younger survivors (under 16 years) in a trauma-informed and creative way throughout the process. Given the constraints we took the ethical decision to work with 16–21-year-olds.

From an equity perspective, we did not reach as many minoritized survivors as we hoped (in particular around disability, migrant status and experience of care), and our demographics questionnaire was too brief to capture the range of intersecting identities participants may have had. We were constrained by time and funding however in the future we aim to proactively include a wider range of survivor groups and consider how we can collect more detailed demographics that are not overly intrusive. Nevertheless, survivor participants in the final consensus workshop shared aspects of their identity around being neurodivergent or LGBTQ+ and practitioners involved had extensive experience of working with marginalized groups.

While the study aimed to ensure the relevance of the DA-COS for the international research and practice community, it was funded to focus on the UK context. The literature and OMIs we reviewed were international in scope, however, further work is needed to adapt the DA-COS for wider use and transferability to different contexts.

## 5 Conclusion

This study resulted in the identification and provisional recommendation of the CAFADA Wellbeing and Safety tool to capture the outcomes of *family relationships* and *feelings of safety*. The study was unable to identify a suitable OMI to capture *freedom to go about daily life*; further research is needed to develop a tool to capture this survivor-identified outcome. Successful implementation of the DA-COS will require trauma-informed guidance, practical integration and implementation strategies for services and research, and ongoing monitoring. However, the selection of OMIs for four of the five outcomes within the DA-COS marks a significant step toward more efficiently evaluating child and family DA interventions.

## Data Availability

The original contributions presented in the study are included in the article/Supplementary material, further inquiries can be directed to the corresponding author.

## Footnotes

1 See Mural (2025) Sync up. Speed up. Stand out. Available at: https://www.mural.co

2 CAFADA (n.d.) Co-developing well-being and safety measures. Available at: https://cafada.stir.ac.uk/wp-content/uploads/2024/09/Timepoint-1-women-survey-workbook.pdf

3 CAFADA, UKRI and University of Sterling (n.d.) Wellbeing and safety survey. Available at: https://cafada.stir.ac.uk/wp-content/uploads/2024/09/Timepoint-1-women-survey-workbook.pdf

4 CAFADA, UKRI and University of Sterling (n.d.) Wellbeing and safety survey. Available at: https://cafada.stir.ac.uk/outputs-and-knowledge-exchange/codeveloping-measures
