# Supplementary material for: Identifying and selecting outcome measures for the children and families domestic abuse core outcome set
Source: Front Sociol. 2026 Feb 18;11:1680919. doi: 10.3389/fsoc.2026.1680919 (PMC12956635; doi:10.3389/fsoc.2026.1680919)
Supplement: Supplementary file 1 [file Data_Sheet_1.pdf]

Supplementary Material

Contents

1 Lived Experience Advisory Group feedback and actions completed 3

1.1 Consultation 1: Recruitment and preparation for workshops 3

1.2 Consultation 2: Feedback on the final shortlist of tools prior to the consensus workshop 4

2 Expert advisory group feedback and actions taken by the research team 6

3 Participant characteristics from all workshops 8

4 Aggregated participant demographic data 10

4.1 Stage C. Acceptability workshop demographic data 10

4.2 Stage D. Survivor feedback and consensus workshops demographic data 10

5 Methodology for the systematic rapid review of reviews 11

6 Eligibility criteria for rapid review of reviews 12

7 Search Strategy for rapid review of reviews 14

8 Methodology for the rapid systematic review of the grey literature 17

9 Eligibility criteria for grey literature searches 18

10 List of websites screened in grey literature searches 19

11 Search terms used for grey literature searches 22

12 Approach used to conduct targeted literature searches 23

13 Flowchart of papers and OMIs identified from all sources 24

14 COSMIN Risk of Bias Checklist 25

15 COSMIN Interpretability and Feasibility Checklist 26

16 Steering Group Checklist 27

17 Practitioner Group Checklist 28

18 Survivor Checklist 29

19 Extract from the measurement information sheet of the multi-participant workshop pre-workshop pack 30

20 Protocol Deviations 38

21 Flowchart of OMIs identified, included, and excluded across all stages 39

22 Core outcome definitions 40

23 Top votes comments from concept workshops 41

24 Thematic synthesis of concept workshop comments 45

25 List of measurement tools included at Stage 2 51

26 List of shortlisted measurement tools for Stage 3 56

27 List of excluded measurement tools for Stage 3 58

28 Description of shortlisted tools 60

29 Acceptability workshop votes on final shortlisted tools progressing to the consensus workshop 63

30 Acceptability workshop comments on final shortlisted tools 64

31 Acceptability workshop votes for excluded tools 72

32 Acceptability workshop comments on excluded tools 73

33 Survivor feedback workshop summary 85

34 Comments for the provisionally recommended measurement tools 93

35 Suggested considerations per recommended tool 96

36 Research team reflexivity statement 98

1 Lived Experience Advisory Group feedback and actions completed

VOICES (n = 7) served as a survivor advisory group at multiple stages. They were first consulted about the recruitment and management of survivor participants and informed on the facilitation of workshops to ensure they were trauma-informed. Second, they provided feedback on the final shortlist of eight tools prior to the consensus workshops; their comments were distributed within the pre-workshop pack (see supplementary material 20). Some members of the advisory group also participated within the consensus workshop.

1.1 Consultation 1: Recruitment and preparation for workshops

Feedback from VOICES	Actions taken by the research team
Discussion around making the workshops more accessible to young people:<br>• Ensure workshops are as accessible and flexible as possible for young people so the workshops are not overwhelming<br>• Be mindful that the young person may still be in contact with the person that harms and this can impact how comfortable they will feel in group workshops	The research team adopted the comments by organising all young people interactions through a young people's coordinator to ensure workshops were accessible and flexible for this participant group
Discussion around the importance of a parent participant group:<br>• Parent–child dyad is fundamental for the delivery of interventions and for inducing change over time<br>• This group could provide a dual perspective (their DA experience and witnessing how children navigate their experience)<br>• Parent participant group can act as a proxy for younger children, providing their perspective as they cannot participate in this project	The research team recruited from SafeLives Pioneers, Refuge Survivor Panel, and VOICES at the DAC to create a parent participant group who provided feedback and voted for the recommended measurement tools at the consensus workshop
Discussion around integrating participant groups during the consensus workshop:<br>• Setting clear expectations and house-keeping rules to ensure everyone feels safe<br>• Professionals should sign a trauma-informed principles document<br>• The research team should ensure there are pre- and post- workshop check-ins for lived-experience participants<br>• Ensure that there is no mixing of parent and child groups (if recruiting from the same organization)	Prior to attending the consensus workshop, all participants signed a series of documents outlining trauma-informed principles that should be followed during the workshop discussions.<br><br>The research team organised multiparticipant workshop groups prior to the event to ensure no inappropriate mixing of participants e.g. related parents and children were not in the same group.

	Briefing workshops were held with all survivors prior to the consensus workshop and post-workshop check-ins were offered to survivors through the workshop counsellor. This was in line with the University of Sussex's safeguarding policy
The young person's advertisement and terms of reference was reviewed, and the following changes were recommended:<br>• Use of plain language<br>• Condensing the text	The research team implemented all comments made by the advisory group by reviewing the language and formatting to improve readability

1.2 Consultation 2: Feedback on the final shortlist of tools prior to the consensus workshop

Measurement tool	Outcome	VOICES feedback
1A: CAFADA Wellbeing and Safety – Relationships Subscale	Family Relationships	• Many items need revision to remove ambiguity or separating into individual constructs.<br>• The child measure doesn't distinguish between positive/negative relationships.<br>• Use of gendered language is problematic
1B: Medical Outcomes Study – Social Support Survey	Family Relationships	• The items were not triggering/problematic.<br>• The language is inaccessible for children to comprehend.<br>• The team recommended a subscale adopted within domestic abuse literature; VOICES preferred the whole tool
1C: Space for Action Scale – Family & Friends; Communities Subscales	Family Relationships	• Liked the reference to the wider community and specifically organizations/external agencies.<br>• Disliked the timeframe for questioning and its impact on the item wording
2A: CAFADA Wellbeing and Safety – Feeling Support Subscale	Feelings of Safety	• Some items were ambiguous or needed separating.<br>• The child version contained items that were inaccessible to children – e.g. would a child know about the support

		their mother or siblings have access to or need?<br>• Different contexts were not considered – e.g. court systems.<br>• Failed to capture how feeling safe is relative – e.g. feeling safer
2B: Roadmap (UCLAN) – Your Safety Subscale	Feelings of Safety	• Different types of safety explored – e.g. online safety.<br>• While the timeframe was suitable for services, it was recommended that this should be revised if implemented in the DA-COS
2C: WHOQOL-100 – Safety Subscale	Feelings of Safety	• Language was minimizing– e.g. "worry" has been used in an unsympathetic way by professionals.<br>• Visually really unappealing and unclear<br>• Disliked the different word prompts for each Likert scale
3A: Space for Action – Help-seeking; Competence; Finance Subscales	Freedom to go about daily life	• Disliked the term "competence" as incompetence is used in instances of coercive control.<br>• Lack of clarity around the timeframe
3B: State Optimism Measure	Freedom to go about daily life	• Felt repetitive and circumstantial.<br>• Optimism may be inappropriate in certain contexts – e.g. at early stages of recovery, you may not have expectations about things going well.<br>• Items need better framing to allow for meaningful exploration in a domestic abuse context

2 Expert advisory group feedback and actions taken by the research team

The expert advisory group is comprised of researchers and practitioners (n = 9) with specialisms in domestic violence and abuse as well as outcomes measurement and measurement validation. For this project, the expert advisory group met twice virtually, and their feedback is described below.

Stage	Feedback from expert advisory group	Actions taken by the Research Team
Stage 1: Overview of the project and considerations going forward.<br><br>(Tuesday 1 October 2024)	• The expert advisory group liked the idea that 'freedom to go about daily life' was framed as a feeling and 'feelings of safety' was framed as only being defined by the person experiencing this feeling. This resonates with DA service provision training. Critically, the research team needs to disentangle the overlap between 'feelings of safety' and 'freedom to go about daily life.'<br>• Recommendation to include community safety within the search for the 'feelings of safety' literature.<br>• Oxford Positive Self-Scale was recommended for consideration for the outcome 'freedom to go about daily life.'<br>• Consideration is needed for tools capturing the person who harms or babies/unborn children.<br>• Issues with the definition of 'family relationships' – care experienced children or kinship.	• The research team worked to disentangle the two outcomes. The team presented their initial rationale for overcoming this issue:<br>o Two subscales could be identified and used in tandem.<br>o Freedom could be viewed as a long-term outcome.<br>• Community safety was screened for with tools that could map to feelings of safety.<br>• Oxford positive self-scale was considered and viewed as inappropriate during previous iterations; however, this tool was revisited.<br>• For unborn babies/babies the research team considered proxy measures<br>• All tools, including those capturing the person that harms and babies/unborn babies,

Stage	Feedback from expert advisory group	Actions taken by the Research Team
	• Comments on defensive reporting (especially early within an intervention)	have been considered (limitations focus on the lack of tools/limited evidence base)
Stage 2: Overview of the project and feedback on the tools<br><br>(Thursday 6 February 2025)	• CAFADA wellbeing and safety is not appropriate for use with perinatal families.<br>• Free text box is popular; however, what is the feasibility and acceptability of processing this?<br>• Concern regarding what gets measured gets done.<br>• Recommendations to ensure the measure is used by services and not just within trials/research data:<br>o Strong guidance is needed to ensure tool is not used just for screening – needs to keep alignment with trauma informed principles.<br>• Possible unintended consequences need to be identified and considered within the trauma informed guidance.<br>• UK Trauma Council (UKTC) – interested in formulating guidance.<br>• Considerations focused on ensuring monitoring data and COS align	The research team will use these comments to provide context and considerations to the research findings within the report and wider uses of the DA-COS

3 Participant characteristics from all workshops

The below table documents the number of participants, and their respective organisations/ affiliations, who participated in each workshop across this project.

	Survivors	Practitioners	Academics
**Stage A:** Concept workshops held online from 10 September to 2 October 2024	Five young people attended from SafeLives' Changemakers	Five practitioners attended from:<br>• Women's Aid<br>• SafeLives<br>• Solace Women's Aid<br>• Southall Black Sisters<br>• Refuge<br>• Acorn Project	Four academics attended from:<br>• Public Health Wales (EU Definition Network)<br>• University Of Central Lancashire<br>• University Of Edinburgh<br>• City, University of London
**Stage C:** Briefing and acceptability workshops held online from 11 November to 16 December 2024	Four young people attended from SafeLives' Changemakers*	Five practitioners attended from:<br>• Women's Aid<br>• SafeLives<br>• Solace Women's Aid<br>• Refuge<br>• Acorn Project	Seven academics attended from:<br>• Public Health Wales (EU Definition Network)<br>• University Of Central Lancashire<br>• University Of Edinburgh<br>• City, University of London<br>• Independent Consultant<br>• Barnardo's
**Stage D:** Survivor feedback and consensus workshops held online	Five survivors attended the survivor feedback sessions from:<br>• Four young people from	Ten practitioners and commissioners attended from:<br>• Women's Aid<br>• SafeLives<br>• Refuge	Eight academics attended from:<br>• City, University of London

from 16 to 28 January 2025	SafeLives' Changemakers<br>• One adult from SafeLives' Pioneers<br>Eleven adult survivors attended the consensus workshops from:<br>• VOICES Charity<br>• VOICES at the DAC<br>• Refuge Survivor Panel	• Solace<br>• Southall Black Sisters<br>• Acorn<br>• For Baby's Sake<br>• Imkaan<br>• DRIVE<br>• Northumberland	• Public Health Wales (EU definition network)<br>• Independent Consultant<br>• University of Edinburgh<br>• University of Central Lancashire<br>• Barnardo's<br>• Cordis Bright<br>• University of Warwick

* One participant within the Changemakers group withdrew from the study between workshops

4 Aggregated participant demographic data

The below tables reflect the aggregated demographic information of participants involved in the acceptability and consensus workshops. The aggregation of this data served to preserve the anonymity of participants. One participant did not provide their demographic data.

4.1 Stage C. Acceptability workshop demographic data

Demographics		n	(%)
**Age**	16–24	3	20.0
	25–34	2	13.3
	35–44	5	33.3
	45–64	5	33.3
**Gender**	Female	15	100
**Ethnicity**	Asian or Asian British (Indian, Pakistani, Bangladeshi, Chinese, any other Asian background)	3	20.0
	Black, African, Caribbean, Black British, or any other Black background)	0	0
	Mixed/multiple ethnic groups or other ethnic groups	2	13.3
	White (English, Welsh, Scottish, Northern Irish, British, Irish, Gypsy or Irish Traveller, Roma, or any other White background)	10	66.7

4.2 Stage D. Survivor feedback and consensus workshops demographic data

Demographics		n	(%)
**Age**	16–24	4	12.1
	25–34	5	15.2
	35–44	9	27.2
	45–54	10	30.3
	55–64	5	15.2
**Gender**	Female	33	100
**Ethnicity**	Asian or Asian British (Indian, Pakistani, Bangladeshi, Chinese, any other Asian background)	14	42.4
	Black, African, Caribbean, Black British, or any other Black background)	3	9.1
	Mixed/Multiple ethnic groups or other ethnic groups	2	6.1
	White (English, Welsh, Scottish, Northern Irish, British, Irish, Gypsy or Irish Traveller, Roma, or any other White background)	14	42.4

5 Methodology for the systematic rapid review of reviews

First, a rapid systematic review of reviews was conducted which screened the academic literature. Search terms relating to domestic abuse interventions for children and their families were inputted into five databases (Cochrane Library, Embase, Medline, PsycINFO and Web of Science) to identify relevant systematic reviews published from May 2019 until July 2024. The inclusion criteria used to screen the literature included: 1) children and their families with experience or at risk of experiencing domestic abuse, 2) interventions or services that specifically targeted survivors of domestic abuse, with at least one child or family outcome measured, 3) interventions with any comparison group, 4) any outcome which reflected the child, the caregiving environment or material deprivation and 5) peer-reviewed systematic reviews of experimental or quasi-experimental studies. (See supplementary material 6 for the eligibility criteria and supplementary material 7 for the search strategy)

The literature underwent both title and abstract screening and full text screening and 5% and 17 of exclusions were independently double screened as a consistency check at the respective stages. At both stages, the research team discussed and resolved all discrepancies. Two researchers extrapolated individual studies and outcome measurement instruments (OMIs) from included systematic reviews and, to mitigate duplications, cross-checked measures against a longlist of candidate OMIs generated from the Home Office work and updated by the CADA project (Powell et al., 2022; Barter et al., in preparation)[1]; novel OMIs were mapped against the core outcomes to determine their relevance and if at least one or more item could map to any aspect of the outcomes, the OMIs progressed to Stage 2.

[1] Barter, C.A., Richardson Foster, H., Howarth, E., Dunk, E., Bracewell, K., Scollay, C., Bedston, E., Humphreys, L., Barlow, C. and Stanley, N. (forthcoming) CADA Evaluation – Children Affected by Domestic Abuse [forthcoming].
Powell, C., Clark, S.E., Downes, L., Feder, G., Fulton, E., Howarth, E., Karia, A., Kimber, M., d'Oliveira, A.F., Shaheen, A. and Vindrola-Padros, C. (2022b) 'A rapid review of outcome measurement tools related to the DVA Core Outcome Set', Epub ahead of print. Available at: https://www.ucl.ac.uk/children-policy-research/sites/children_policy_research/files/dva_measures_ho_report_06122022.pdf.

6 Eligibility criteria for rapid review of reviews

The following PICOS[2] criteria were implemented to identify relevant measurement tools from the academic literature.

PICOS criteria	Inclusion criteria	Exclusion criteria
**Population**	Children or families with children with/at risk of DA exposure. This includes unborn children, children (0–18 years) designated as a victim or witness. Any adult family members who have a parenting role, whether designated as perpetrator, victim, witness, or household member. Adults or children could either be the primary study population of interest or form a subgroup in a wider study population	Any population that has no experience of domestic violence and abuse between parents; this includes elder abuse, sibling abuse, child-to-parent abuse, dating violence, or child maltreatment. Adult-only populations, or groups where a parental perspective is not explored, were also excluded as part of our criteria.
**Intervention/ exposure**	Any intervention or service where experience of or increased risk of experiencing DA is a criterion for being offered the service OR DA is measured as an exposure or outcome of interest AND at least one child- or family-level outcome is measured (affects the family/household unit). Studies must include evaluation of a defined activity/program and evaluation of hypothesized effect. Interventions may be delivered to any family members individually or in groups. Any duration of interventions will be included	Universal interventions that do not specifically target children/families at risk of DA; target interventions that do not measure any child- or family-level outcomes or focus on elder abuse, sibling abuse, child perpetration of DA where participants have not been identified as exposed to DA.

[2] The PICO criteria reference an evidence-based framework use to formulate research questions and consists of the population, intervention or exposure, comparison, outcomes; additions to this criteria includes defining the study design of eligible literature (Hosseini, M.-S., Jahanshahlou, F., Akbarzadeh, M.A., Zarei, M. and Vaez-Gharamaleki, Y. (2024) ‘Formulating research questions for evidence-based studies’, Journal of Medicine, Surgery, and Public Health, 2, p. 100046. Available at: https://doi.org/10.1016/j.glmedi.2023.100046.).

**Comparator**	Any control comparison group/period with participants receiving no care, treatment as usual, or any other treatment.	No exclusions were placed on this criterion
**Outcome**	Any outcome reflecting the child, caregiving environment, or material deprivation. Outcomes can be reported by professionals, the child, parent, or other family members and they can be retrospective or prospective. Outcomes can be ending points, surrogate markers for endpoints, or intermediate outcomes. No maximum follow-up is required.	No exclusions were placed on this criterion.
**Study design**	Peer-reviewed systematic reviews of controlled or quasi-experimental comparator intervention studies with or without randomization. This needs to search an electronic database and have a structured search strategy published since May 2019. Papers must be in English, but no restrictions on country. Individual studies must include DA in one of the following ways:<br>a. entry to the intervention is determined by experience, perpetration, or identified by researcher/practitioner/participant as at risk of DA.<br>b. subgroup analysis is carried out by participants with experiences/at risk of DA.<br>c. DA is measured as an exposure (retrospective/prospectively)	Non-peer-reviewed studies, qualitative studies, general literature reviews, protocols, case reports, cross-sectional studies, general discussion papers, letters, commentaries, book chapters, conference papers, theses and dissertations

7 Search Strategy for rapid review of reviews

The below table documents the search strategy used, and the number of systematic reviews identified, for each database searched.

Database	Search string	Total
**Web of Science**	1. TS= ((abus* or violen* or coerci* or batter* or non-accidental injur* or aggress* or anger or victimi?ation) AND (Partner or spouse or famil* or wife or wives or wom*n or maternal or parent* or batter* or interpar* or domestic or intimate partner or household or marital or couple* or marital or m*n or husband* or victim or perpetrator or witness* or experienc* or expos* or risk or "living with" or vulnerable or child or infant or unborn or f?etus or young person or teenage* or adol*))<br>2. TS= ((systematic NEAR/2 review) OR (systematic NEAR/2 overview) or "review of reviews")<br>3. 1 and 2 AND LANGUAGE: (English) Indexes=SCI-EXPANDED, SSCI, A&HCI, ESCI; Timespan=Last 5 years	5,440
**Cochrane Library**	((MeSH descriptor [domestic violence] explore all trees) OR (MeSH descriptor [Intimate partner violence] explore all trees) OR (MeSH descriptor [Gender-Based violence] explore all trees) OR (MeSH descriptor [Battered Women] explore all trees) OR ((MeSH descriptor [Exposure to violence] explore all trees) AND (MeSH descriptor [Child] explore all trees)) OR MeSH descriptor [Exposure to violence] explore all trees) AND (MeSH descriptor [Women] explore all trees)) OR ((abus* or violen* or coerci* or batter* or non-accidental injur* or aggress* or anger or victimi?ation) NEAR (Partner or spouse or famil* or wife or wives or wom*n or maternal or parent* or batter* or interpar* or domestic or intimate partner or household or marital or couple* or marital or m*n or husband* or victim or perpetrator or witness* or experienc* or expos* or risk or "living with" or vulnerable or child or infant or unborn or f?etus or young person or teenage* or adol*)) AND date limitation from May 2019 until current	10
**Embase**	((Exp domestic violence/) OR (Exp gender-based violence/) OR ((Exposure to violence/) AND (Exp Child/)) OR ((Exposure to violence/) AND (Exp female/)) OR ((abus* or violen* or coerci* or batter* or non-accidental injur* or aggress* or anger or victimi?ation) adj2 (Partner or spouse or famil* or wife or wives or wom*n or maternal or parent* or batter* or interpar* or domestic or intimate partner or household or marital or couple* or marital or	1,251

	m?n or husband* or victim or perpetrator or witness* or experienc* or expos* or risk or living with or vulnerable or child or infant or unborn or f?etus or young person or teenage* or adol*)).mp.) AND ((((Exp review/) OR ((literature adj3 review$).ti,ab.) OR (Exp meta analysis/) OR (Exp "systematic review"/)) AND ((medline or medlars or embase or pubmed or cinahl or amed or psychlit or psyclit or psychinfo or psycinfo or scisearch or cochrane).ti,ab.) OR (RETRACTED ARTICLE/))) OR ((systematic$ adj2 (review$ or overview)).ti,ab.) OR ((meta?anal$ or meta anal$ or meta-anal$ or metaanal$ or metanal$).ti,ab.)) AND ((english language and yr="2019 -Current")	
**Medline**	((Exp domestic violence/) OR (Exp Intimate partner violence/) OR (Exp gender-based violence/) OR (Exp battered violence/) OR ((Exposure to violence/) AND (Exp child/)) OR ((Exposure to violence/) AND (Exp women/)) OR (((abus* or violen* or coerci* or batter* or non-accidental injur* or aggress* or anger or victimi?ation) adj2 (Partner or spouse or famil* or wife or wives or wom*n or maternal or parent* or batter* or interpar* or domestic or intimate partner or household or marital or couple* or marital or m*n or husband* or victim or perpetrator or witness* or experienc* or expos* or risk or living with or vulnerable or child or infant or unborn or f?etus or young person or teenage* or adol*)).mp.) AND (Exp "systematic review"/) OR ((Review.pt.) AND (((medline or medlars or embase or pubmed or cochrane).tw,sh.) OR ((scisearch or psychinfo or psycinfo).tw,sh.) OR ((psychlit or psyclit).tw,sh.) OR (cinahl.tw,sh.) OR (((hand adj2 search$) or (manual$ adj2 search$)).tw,sh.) OR ((electronic database$ or bibliographic database$ or computeri?ed database$ or online database$).tw,sh.) OR ((pooling or pooled or mantel haenszel).tw,sh.) OR ((peto or dersimonian or der simonian or fixed effect).tw,sh.) OR ((retraction of publication or retracted publication).pt.)) OR ((meta-analysis.pt.) OR (meta-analysis.sh.) OR ((meta-analys$ or meta analys$ or metaanalys$).tw,sh.) OR ((systematic$ adj5 review$).tw,sh.) OR ((systematic$ adj5 overview$).tw,sh.) OR ((quantitativ$ adj5 review$).tw,sh.) OR ((quantitativ$ adj5 overview$).tw,sh.) OR ((quantitativ$ adj5 synthesis$).tw,sh.) OR ((methodologic$ adj5 review$).tw,sh) OR ((methodologic$ adj5 overview$).tw,sh.) OR ((integrative research review$ or research integration).tw.))) AND (english language AND yr="2019 -Current")	1,538
**PsycInfo**	((((Domestic violence/) OR (Exp intimate partner violence/) OR (Exp battered females/) OR ((Exposure to violence/) AND	1,895

	(Child.mp)) OR ((Exposure to violence/) AND (Exp human females/))) OR (((abus* or violen* or coerci* or batter* or non-accidental injur* or aggress* or anger or victimi?ation) adj2 (Partner or spouse or famil* or wife or wives or wom?n or maternal or parent* or batter* or interpar* or domestic or intimate partner or household or marital or couple* or marital or m?n or husband* or victim or perpetrator or witness* or experienc* or expos* or risk or living with or vulnerable or child or infant or unborn or f?etus or young person or teenage* or adol*)).mp.)) AND (((Exp literature review/) OR ((Review$.mp) AND (((medline or medlars or embase or pubmed or cochrane).mp) OR ((scisearch or psychinfo or psycinfo).mp) OR ((psychlit or psyclit).mp) OR (cinahl.mp) OR (((hand adj2 search$) or (manual$ adj2 search$)).mp) OR ((electronic database$ or bibliographic database$ or computeri?ed database$ or online database$).mp) OR ((pooling or pooled or mantel haenszel).mp) OR ((peto or dersimonian or der simonian or fixed effect).mp) OR ((retraction of publication or retracted publication).mp))) OR ((Exp meta analysis/) OR ((meta-analys$ or meta analys$ or metaanalys$).mp) OR ((systematic$ adj5 overview$).mp) OR ((quantitativ$ adj5 overview$).mp) OR ((quantitativ$ adj5 synthesis$).mp) OR ((methodologic$ adj5 overview$).mp) OR ((research integration).mp)))) AND (english language and yr="2019 -Current")

8 Methodology for the rapid systematic review of the grey literature

A second rapid review was conducted the screened the grey literature and websites of key domestic abuse organisation. Again, search terms were used to identify child and family-focused interventions and outcomes in publications available from 2021 until August 2024. Mirroring the academic rapid review, candidate OMIs were extrapolated from relevant literature and were mapped against the core outcomes. If one or more items appropriately mapped, the OMI progressed to stage 2. (see supplementary material 9 for the eligibility criteria, supplementary material 10 for the list of websites searched and supplementary material 11 for the search strategy).

9 Eligibility criteria for grey literature searches

The following PICOS criteria were implemented to identify relevant measurement tools from the grey literature.

PICOS criteria	Inclusion criteria	Exclusion criteria
**Population**	Children or families with children with/at risk of DA exposure. This includes unborn children, children (0–18 years) designated as a victim or witness. Any adult family members who have a parenting role, whether designated as perpetrator, victim, witness, or household member. Adults or children could either be the primary study population of interest or form a subgroup in a wider study population.	Any population that has no experience of domestic violence and abuse between parents; this includes elder abuse, sibling abuse, child-to-parent abuse, dating violence, or child maltreatment. Adult-only populations, or groups where a parental perspective is not explored, were also excluded as part of our criteria
**Intervention/ exposure**	No criteria were set	No criteria were set
**Comparator**	No criteria were set	No criteria were set
**Outcome**	Any outcome reflecting the child, caregiving environment, or material deprivation. Outcomes can be reported by professionals, the child, parent, or other family members and they can be retrospective or prospective. Outcomes can be end points, surrogate markers for endpoints, or intermediate outcomes. No maximum follow-up is required.	Measurement tools that do not measure any child- or family-level outcomes or focus on elder abuse, sibling abuse, dating violence, or child perpetration of DA where participants have not been identified as exposed to DA
**Study design**	Published since May 2021. Publications must be in English, but no restrictions on country	Publications that were published prior to 2021 and were not written in English were excluded

10 List of websites screened in grey literature searches

The below table documents the 72 websites searched and the dates these searches were conducted as part of the grey literature searches. Both NICE evidence repository and Open Grey closed prior to the commencement of this literature search.

Organization	Website	Date searched
SafeLives	https://safelives.org.uk	08/08/2024
Respect	https://www.respect.org.uk	08/08/2024
Nice Evidence Search	https://www.nice.org.uk/about/what-we-do/evidence-services	08/08/2024
Open Grey	https://www.greynet.org/opengreyrepository.html	08/08/2024
Refuge	https://refuge.org.uk	09/08/2024
Foundations	https://foundations.org.uk	09/08/2024
Advance	https://www.advancecharity.org.uk	13/08/2024
Imkaan	https://www.imkaan.org.uk	14/08/2024
IRISi	https://irisi.org/#	14/08/2024
VOICES Charity	https://www.voicescharity.org	15/08/2024
NSPCC	https://www.nspcc.org.uk	19/08/2024
Barnardo’s	https://www.barnardos.org.uk	20/08/2024
Victim Support	https://www.victimsupport.org.uk	22/08/2024
Standing Together	https://www.standingtogether.org.uk	22/08/2024
Woman’s Trust	https://womanstrust.org.uk	22/08/2024
DVIP (Domestic Violence Intervention Programme)	https://dvip.org	22/08/2024
Nia	https://niaendingviolence.org.uk	22/08/2024
The Havens	https://thehavens.org.uk	22/08/2024
ManKind Initiative	https://mankind.org.uk	23/08/2024
Everyman Project	https://justiceinnovation.org/project/everyman-project	23/08/2024
NCDV	https://www.ncdv.org.uk	23/08/2024
Galop	https://galop.org.uk	23/08/2024
LAWA	https://lawadv.org.uk	23/08/2024
IDAS	https://idas.org.uk	23/08/2024
Your Sanctuary	https://www.yoursanctuary.org.uk	27/08/2024
Advocacy After Fatal Domestic Abuse (AAFDA)	https://aafda.org.uk	27/08/2024
Aurora New Dawn	https://www.aurorand.org.uk	27/08/2024
My Sister’s Place	https://mysistersplace.org.uk	27/08/2024

Early Intervention Foundation	https://www.eif.org.uk	27/08/2024
NatCen	https://natcen.ac.uk	27/08/2024
RCGP	https://www.rcgp.org.uk	27/08/2024
RCN	https://www.rcn.org.uk	27/08/2024
RCM	https://www.rcm.org.uk	27/08/2024
NICE	https://www.nice.org.uk	27/08/2024
BPS	https://www.bps.org.uk	27/08/2024
IHV	https://ihv.org.uk	27/08/2024
Working Together	https://www.workingtogetheronline.co.uk	27/08/2024
What Works For Children's Social Care	https://whatworks-csc.org.uk	27/08/2024
Joseph Rowntree Foundation	https://www.jrf.org.uk	27/08/2024
The National Lottery Community Fund, previously known as Big Lottery	https://www.tnlcommunityfund.org.uk	27/08/2024
AVA	https://avaproject.org	27/08/2024
The Childhood Trust	https://www.childhoodtrust.org.uk	28/08/2024
What Works Network	https://www.gov.uk/guidance/what-works-network	28/08/2024
Gov.UK	https://www.gov.uk/crime-justice-and-law/domestic-violence	28/08/2024
Comic Relief	https://www.comicrelief.com	28/08/2024
WHO	https://www.who.int	28/08/2024
UNICEF	https://www.unicef.org	28/08/2024
Women's Aid	https://www.womensaid.org.uk	28/08/2024
Public Health England	https://www.gov.uk/government/organisations/public-health-england	28/08/2024
Public Health Scotland	https://publichealthscotland.scot	28/08/2024
Public Health Wales	https://phw.nhs.wales	28/08/2024
Northern Ireland PHA	https://www.publichealth.hscni.net	28/08/2024
Children's Commissioner England	https://www.childrenscommissioner.gov.uk	29/08/2024
Children's and Young People's Commissioner Scotland	https://www.cypcs.org.uk	29/08/2024

Children's Commissioner for Wales	https://www.childcomwales.org.uk	29/08/2024
Northern Ireland Children's Commission	https://www.niccy.org	29/08/2024
UK College of Policing	https://www.college.police.uk	29/08/2024
Research In Practice	https://www.researchinpractice.org.uk	29/08/2024
For Baby's Sake previously known as The Stefanou Foundation	http://www.stefanoufoundation.org/https://forbabyssake.org.uk	29/08/2024
Work with Perpetrators of Domestic Violence (Europe)	https://www.work-with-perpetrators.eu	09/09/2024
Hestia	https://www.hestia.org	10/09/2024
Domestic Violence Evidence Project	https://www.dvevidenceproject.org	10/09/2024
Asian Women's Resource Centre	https://www.asianwomencentre.org.uk	10/09/2024
Global Network of Women's Shelters	https://gnws.org	13/09/2024
VAMHN	https://www.vamhn.co.uk	13/09/2024
Domestic Abuse Commissioner	https://domesticabusecommissioner.uk	13/09/2024
VAWNET	https://vawnet.org	13/09/2024
End Violence Against Women	https://www.endviolenceagainstwomen.org.uk	15/09/2024
DAPHNE	https://eucpn.org/document/daphne	15/09/2024
WAVE	https://www.wave-network.org	15/09/2024
Youth Endowment Fund	https://youthendowmentfund.org.uk	16/09/2024
Agenda	https://www.agendaalliance.org	20/09/2024

11 Search terms used for grey literature searches

Websites were searched using the below key phrases to identify literature relevant to the inclusion criteria. Literature from websites was then screened for the below terms to determine whether the literature included details on measurement tools that met the inclusion criteria.

Website tool bar search phrases	Website tab pages searched	Key words searched in publications
“measurement tool”	Look for “Reports/publications/resources” page	“measure”
“measure”	Look for “Programmes/interventions/ projects/services” page	“framework”
“domestic violence”		“measurement”
“domestic abuse”		“survey”
		“tool”
		“scale”
		“instrument”
		“outcome”
		“evaluation”

12 Approach used to conduct targeted literature searches

The following search string and terms were used to identify additional relevant measurement tools that mapped to the remaining three core outcomes. Each search was conducted on PubMed and Google Scholar to capture both academic and grey literature publications, with the top 50 papers screened for relevant tools.

Themes identified from concept workshops	Boolean operator used	Measurement tool key words
Aspirations	AND	Measure
Community safety		Tool
Family functioning		Index
Feelings of trauma		Survey
Future		Questionnaire
Hope		Outcome
Hypervigilance		Scale
Self-actualization		
Stalking		
Stress		

13 Flowchart of papers and OMIs identified from all sources

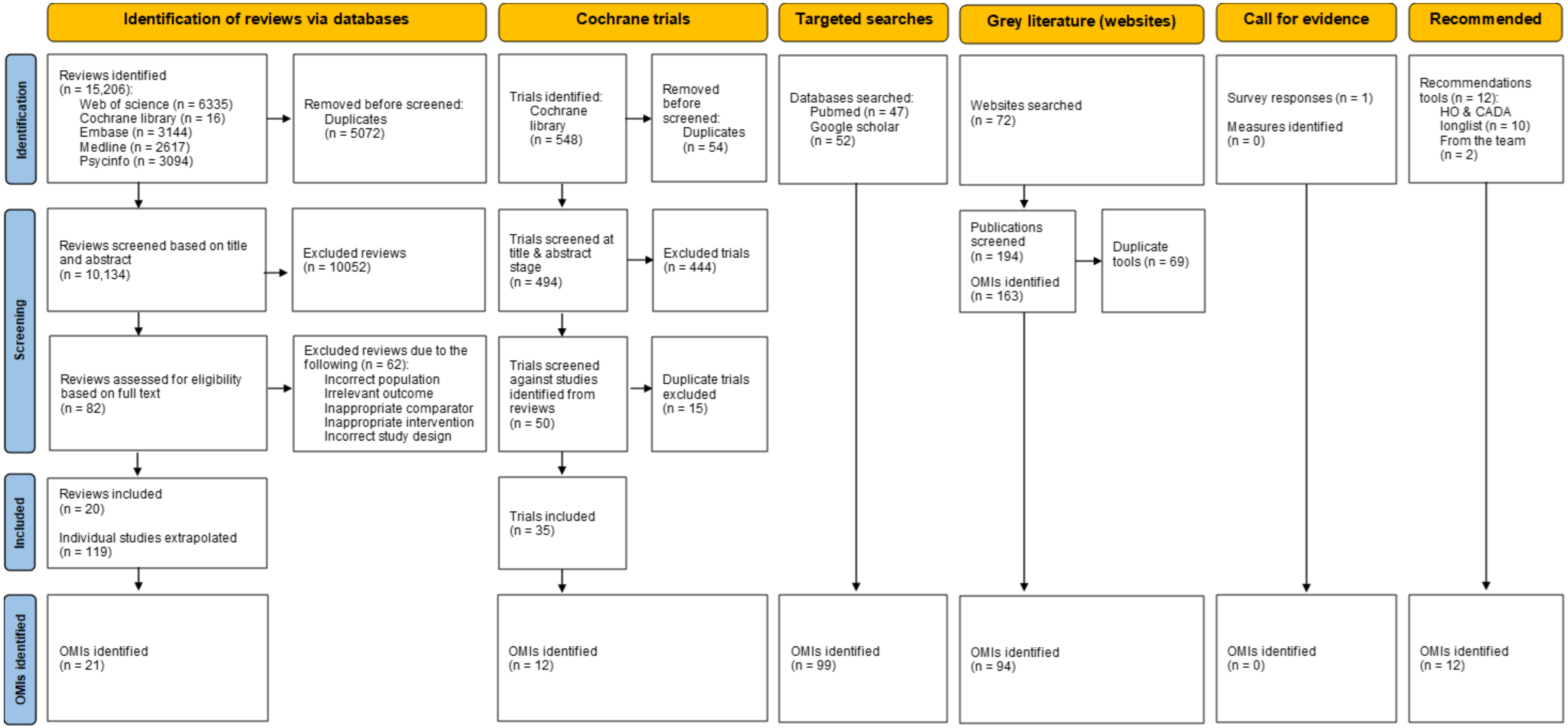

14 COSMIN Risk of Bias Checklist

Subdomain	Criteria – COS Risk of Bias
Content Validity including PROM development	1. Is a clear description provided of the construct to be measured?<br>2. Is the origin of the construct clear: was a theory, conceptual framework or disease model used or clear rationale provided to define the construct to be measured?<br>3. Is a clear description provided of the target population for which the PROM was developed?<br>4. Is a clear description provided of the context of use?<br>5. Was the PROM development study performed in a sample representing the target population for which the PROM was developed?<br>6. PROM development - What were the methods that were used to develop the tool?<br>7. Was a cognitive interview study or other pilot test conducted?<br>8. Were participants asked about the comprehensibility of the PROM during the development study?<br>9. Were participants asked about the comprehensiveness of the PROM during the development study?
Internal Structures and Measurement properties	1. Was structural validity assessed? If so, what assessments were done?<br>2. Was internal consistency or reliability assessed? If so, what assessments were done?<br>3. Cross-cultural validity - Has anything been done to translate the measure into other cultures (systematic testing of the contextual concepts)? If so, please describe?<br>4. Was criterion validity assessed? If so, what assessments were done?

15 COSMIN Interpretability and Feasibility Checklist

	Criteria
1.	Qualitative meaning to the tools quantitative scores/ score for clinical cut off?
2.	Patient's required mental and physical ability level - is the tool usable in patients with low mental and physical ability?
3.	Has the tool been validated?
4.	Can the tool be used without regulatory agency's approval?

16 Steering Group Checklist

Subdomain	Criteria – Steering Group
Descriptors	1. What population was this tool originally validated in?<br>2. Does it measure broad or specific outcomes?<br>3. Is the tool self-reported or delivered by a clinician/ practitioner?
Use in evaluative studies	1. Has the tool been used in an interventional and academic setting or interventional only?<br>2. Has the tool been used historically or just a potential use?<br>3. Has the tool been evaluated in a pre- post- framework?<br>4. What population-based study design has the tool been measured in?
Use with diverse populations	1. Is the tool used across multiple sectors or only DA specific sectors?<br>2. Has the tool been used in a different population to test if the contextual and cultural concepts are the same?<br>3. Were contextual and cultural concepts the same?<br>4. Is the tool available in different languages?<br>5. If so which languages?
Psychometric Robustness	1. Are there any tests of reliability?<br>2. Are there any tests of validity?
Time	1. Does it take a short time to deliver the tool?<br>2. Is the instrument short in number of questions?<br>3. Is training required to administer the tool?<br>4. If training is required - does it take a long time?<br>5. If training is required - is refresher training needed?
Cost	1. Is payment required for training?<br>2. Is payment required to use the tool?<br>3. If so, is the program purchased on a one-off basis or more routinely?<br>4. Is additional equipment required to deliver the tool?
Ease	1. Is it straightforward to calculate the score?<br>2. Can the tool administrator use the tool without a PhD or MD?
Data Collection	1. Does the tool try to address problems with literacy?<br>2. Is the measure used for a therapeutic purpose or routine data collection or the evaluation of a service, or a combination of the above?<br>3. Can the outcome data be easily collected routinely or is complex outcome data that needs to be collected in waves?<br>4. Does this measurement tool fit in easily with what is already being collected from the population?

17 Practitioner Group Checklist

Subdomain	Criteria – Practitioner Checklist
Descriptors	1. Does the tool collect overlapping data on any of the outcomes?<br>2. What type of data is collected/questions formatted?
Narrative Approach	1. Does the tool include narrative data collection to understand the context of an individual and their responses?<br>2. Is the tool strengths based?
Structural Inequalities	1. Does the tool attempt to link an individual's identity and history to understand who/what institution the individual may be threatened by?<br>2. Does the tool flag that individuals may not be responding in an open and confident manner due to their context?<br>3. Does the tool flag who the tool should be delivered by (e.g. not police) to ensure the respondent can respond openly?
Using tools with children	1. Does the tool include a section that ensures the children clearly understand their rights?<br>2. Does the tool include a section that ensures the children understand why the information is being collected from them?<br>3. Does the tool capture the perspective of the child? Or an external perspective, such as a system completing an assessment on the child's behalf?
Implementation of tools	1. Does a manual/guideline accompany the tool?<br>2. Do staff need to be assessed on how they use the tool to ensure they are used in the same way?<br>3. Was there more than one person using the tool to assess the individual to prevent unconscious bias?
Feelings of safety	1. Does the tool capture internal, external and relational safety?<br>2. What aspect of safety was measured?<br>3. Does the tool capture a narrative context, which is needed to really understand safety from the perspective of a child?
Freedom to go about daily life	1. Does the tool relate this outcome to the Rights of the Child Convention?<br>2. Has the tool been used in children, as this outcome is mostly measured in women?
Family relationships	1. Have extended family members been accounted for?<br>2. Has the tool asked the child they think is important to them including contacts that aren't relatives?<br>3. Has the tool been used in a parental conflict resolution intervention or in a women centric model intervention, or both?<br>4. Has responsibility for the child been incorporated into the tool?<br>5. Has the tool enabled narrative data collection to understand the complexity of family and extended family relationships?

18 Survivor Checklist

	Criteria
1.	Is the tool strengths-based?
2.	Is the tool sensitive or intrusive?
3.	Does the tool use judgmental language (e.g. toxic relationships?
4.	Does the tool use unsuitable language for DA e.g. couples’, ‘family together’
5.	Does the tool use minimizing language e.g. absolving the perpetrator
6.	Does the tool use inclusive language e.g. around faith or finances?
7.	Has the tool been used in DA study or practice context?

19 Extract from the measurement information sheet of the multi-participant workshop pre-workshop pack

The below tables collated all the information identified at all prior stages (all workshops and data extraction stages) to ensure participants were as informed as possible prior to voting in the consensus workshop. The pack also contained the core outcome definitions (supplementary material 22), which were informed by the concept workshops (supplementary material 23). The pre-workshop pack was created and distributed by the research team 10 days in advance of the consensus workshop.

<table>
<tr><th colspan="6">1A: CAFADA Safety and Wellbeing Scale</th></tr>
<tr><th rowspan="2">Description</th><th rowspan="2">Strengths</th><th rowspan="2">Weaknesses</th><th colspan="3">Weighted scores (%)</th></tr>
<tr><th>Psychometric</th><th>Acceptability</th><th>Overall</th></tr>
<tr><td>Outcomes:
• Family relationships
• Feelings of safety</td><td>From published research:
✓ This questionnaire can be used to explore more than one core outcome – this reduces the number of questionnaires to be completed (responder burden)
✓ This tool was co-developed with DA survivors.
✓ This questionnaire has a free text box for additional/clarifying information</td><td>From published research
✗ This measurement tool is very new and therefore there is no published research telling us about its scientific strengths.
✗ As this is so new, this measure has not been tested and approved for use on a diverse population</td><td rowspan="2">28.75</td><td rowspan="2">74.11</td><td rowspan="2">51.43</td></tr>
<tr><td>Responder:
• Adults – lilac questionnaire
• Children – green questionnaire</td><td>Comments from workshops:
✓ This questionnaire was generally well liked by the Changemakers:
o There is the correct level of detail in the questions.
o The measure is easy to understand.
o Really liked the free text box</td><td>Comments from workshops:
✗ All felt the child’s measure needs to be improved to avoid vagueness – e.g. what do “difficult times” or “important things” mean?
✗ The Changemakers preferred the adult measure and felt other older children/young adults will find the child measure inappropriate.
✗ Changemakers felt questions should be reworded to reflect different family structures – e.g. families without a ‘mum’</td></tr>
</table>

1B: Medical Outcomes Study - Social Support Survey					
**Description**	**Strengths**	**Weaknesses**	**Weighted scores (%)**		
			**Psychometric**	**Acceptability**	**Overall**
**Outcomes:**<br>• **Family relationships**	From published research:<br>✓ This tool comes with a manual/ guidance on the best way to administer the questionnaire.<br>✓ The questionnaire is culturally sensitive as it has been used with people from different cultural backgrounds	From published research:<br>✗ This questionnaire was developed to collect health data and has not been used with interventions or in a therapy setting.<br>✗ This tool was not developed, nor has it been tested or adapted, for use with children/ young people.<br>✗ We cannot say this tool is entirely inclusive as it's unclear if this measure can be used with people with different accessibility needs	61.31	50.6	55.95
**Responder:**<br>• **Adults**	Comments from workshops:<br>✓ The wording of these questions was well liked as this provided sufficient detail, not too vague.<br>✓ The Changemakers liked the questions referenced 'someone' so responders could explore different relationships.<br>✓ The Changemakers liked the frequency scale used	Comments from workshops:<br>✗ All felt question 1 needs rewording or should be removed as it could be distressing and does not provide a lot of useful information.<br>✗ The Changemakers felt the answer scale needed rewording as it's difficult to understand the difference between "a little of the time" and "some of the time"<br>o This could be changed to 'occasionally' and 'regularly.'<br>✗ All disliked questionnaire format as it felt cramped			

1C: Space for Action Scale					
Description	Strengths	Weaknesses	Weighted scores (%)		
			Psychometric	Acceptability	Overall
**Outcomes:**<br>• **Family relationships**<br>• **Freedom to go about daily life**	From published research:<br>✓ This tool maps onto multiple outcomes<br>✓ The questionnaire was developed, alongside survivors, specifically for domestic abuse contexts.<br>✓ The tool has been delivered to people who have experienced poor mental health	From published research:<br>✗ More scientific testing is needed to tell us about the quality of this measure (validity)<br>✗ One example is approving this measure for use with different populations (e.g. with children, or adults from different ethnic, cultural, and religious backgrounds or those with different accessibility needs)	68.77	48.21	58.49
**Responder:**<br>• **Adults**	Comments from workshops:<br>✓ Many liked that the questionnaire included the different topics not covered in other tools:<br>o E.g. wider community as part of family relationships as they are integral and can be used to identify people who are isolated<br>✓ The Changemakers preferred the questions using 'I' and 'me' as this feels more personal	Comments from workshops:<br>✗ Everyone wanted this tool to include a timeframe to frame the statements (e.g. in the past three months) as an alternative to rewording statements to the present tense<br>✗ Many wanted more statements to be included in the friends and family subscale and felt the statements did not provide a lot of useful information for a service/researcher to understand family relationships.<br>✗ All, but specifically Changemakers, felt some statements were too vague – e.g. the terms "enough" or "comfortable" were subjective and difficult to answer.<br>✗ Changemakers felt the 'communities' questions may be difficult for young people as this group tend to be a part of many communities			

<table>
<tr><th colspan="6">2A: CAFADA Safety and Wellbeing Scale</th></tr>
<tr><th rowspan="2">Description</th><th rowspan="2">Strengths</th><th rowspan="2">Weaknesses</th><th colspan="3">Weighted scores (%)</th></tr>
<tr><th>Psychometric</th><th>Acceptability</th><th>Overall</th></tr>
<tr><td>Outcomes:
• Family relationships
• Feelings of safety</td><td>From published research:
✓ This questionnaire can be used to explore more than one core outcome – this reduces the number of questionnaires to be completed (responder burden)
✓ This tool was co-developed with DA survivors.
✓ This questionnaire has a free text box for additional/clarifying information</td><td>From published research
✗ This measurement tool is very new and therefore there is no published research telling us about its scientific strengths.
✗ As this is so new, this measure has not been tested and approved for use on a diverse population</td><td rowspan="2">28.75</td><td rowspan="2">74.11</td><td rowspan="2">51.43</td></tr>
<tr><td>Responder:
• Adults – lilac questionnaire
• Children – green questionnaire</td><td>Comments from workshops:
✓ This questionnaire was generally well liked by the Changemakers:
o They felt the number of questions created a clear picture.
o There is the correct level of detail in the questions.
o The measure is easy to understand.
o Really liked the free text box</td><td>Comments from workshops:
✗ All felt the child's measure needs to be improved to avoid vagueness:
o Some statements should be separated to be easier for children to answer.
o Some topics children may not know about – e.g. my mum and brothers and sisters have the support they need from services and professionals.
✗ Many disliked the gendered language used and felt more inclusive terms should be used.
✗ For feelings of safety, the adult questionnaire could be revised to show that safety as relative</td></tr>
</table>

2B: Roadmap (UCLAN) – Your Safety Subscale					
**Description**	**Strengths**	**Weaknesses**	**Weighted scores (%)**		
			Psychometric	Acceptability	Overall
**Outcomes:**<br>• **Feelings of safety**	From published research:<br>✓ This questionnaire has been used within five DA services in England known as Beacon sites.<br>✓ Survivors were involved in the development of this tool	From published research:<br>× More scientific testing is needed as the tool itself has not been tested but it has been adapted from a scientifically strong questionnaire – e.g. is this tool approved for use in minority groups or those with accessibility needs?	32.34	64.29	48.31
**Responder:**<br>• **Adults**	Comments from workshops:<br>✓ Many liked that the questions reflect different areas of safety.<br>✓ The Changemakers liked that the questionnaire was short and formatted neatly	Comments from workshops:<br>× Everyone wants the timeframe used in this questionnaire to be changed if used in the DA-COS<br>× The Changemakers wanted a free text box to expand on the following topics:<br>o Exploring what safety looks like to the responder to use as a benchmark.<br>o To explore broader topics in more detail such as which sites you feel safe/unsafe on (e.g. TikTok vs Google)<br>× Changemakers wanted questions to be reworded to remove gendered language.<br>× A question like 'it is safe to express my views and opinions' was recommended by the Changemakers to improve the questionnaire			

2C: World Health Organization Quality of Life 100 (WHOQOL-100) – Safety Subscale					
**Description**	**Strengths**	**Weaknesses**	**Weighted scores (%)**		
			Psychometric	Acceptability	Overall
**Outcomes:**<br>• **Feelings of safety**	From published research:<br>✓ Culturally this tool is very inclusive as it has been developed and used in multiple countries and translated into over 30 languages.<br>✓ Some scientific testing has been done on this tool.<br>✓ This tool comes with a manual/guidance to tell us the best way to administer the questionnaire	From published research:<br>✗ The scientific testing on this tool is low because the whole questionnaire is too long, and adapted versions of this questionnaire preferred to be used in research.<br>✗ The questions are not very strengths-based (not worded positively) which is preferred for tools used in DA contexts.<br>✗ The subscale is very short as there are only four questions in the tool that ask about safety	68.45	48.81	58.63
**Responder:**<br>• **Adults**	Comments from workshops:<br>✓ The Changemakers felt the wording of questions meant it is easy to determine if someone feels safe or not<br>✓ Some of the Changemakers liked that it uses different Likert scales as this made the questionnaire feel less repetitive	Comments from workshops:<br>✗ Everyone felt the measure was visually unappealing.<br>✗ Some of the questions need to be reworded or separated:<br>o Question 2: someone may not be aware their environment is objectively unsafe.<br>o Question 3: someone can be secure but feel unsafe so this question should be separated and it could be considered minimising<br>✗ The Changemakers felt, with only four questions, this tool could feel too generic and not very informative<br>✗ Many disliked the different Likert scales used and felt it was unclear			

3A: Space for Action Scale					
**Description**	**Strengths**	**Weaknesses**	**Weighted scores (%)**		
			Psychometric	Acceptability	Overall
**Outcomes:**<br>• **Family relationships**<br>• **Freedom to go about daily life**	From published research:<br>✓ This tool maps onto multiple outcomes<br>✓ The questionnaire was developed, alongside survivors, specifically for domestic abuse contexts.<br>✓ The tool has been delivered to people who have experienced poor mental health	From published research:<br>× More scientific testing is needed to tell us about the quality of this measure (validity)<br>× One example is approving this measure for use with different populations (e.g. with children, or adults from different ethnic, cultural, and religious backgrounds or those with different accessibility needs)	68.77	48.21	58.49
**Responder:**<br>• **Adults**	Comments from workshops:<br>✓ Many liked that the questionnaire includes the different topics not covered in other tools:<br>o E.g. finances and help-seeking as a part of 'freedom' as it covers different types of abuse<br>✓ The Changemakers preferred the questions using 'I' and 'me' as this feels more personal.<br>✓ Many liked the number of questions in this subscale	Comments from workshops:<br>× Everyone wanted this tool to include a timeframe to frame the statements (e.g. in the past three months) as an alternative to rewording statements to the present tense<br>× Some statements, such as 'competence', needed rewording to specifically outline when someone is limited by the DA experience and not because they could not do the task<br>× The Changemakers felt some topics were inappropriate for children to answer – e.g. community or finance/budgeting statements			

3B: State Optimism Measure					
**Description**	**Strengths**	**Weaknesses**	**Weighted scores (%)**		
			Psychometric	Acceptability	Overall
**Outcomes:**<br>• **Freedom to go about daily life**	From published research:<br>✓ Moderate scientific testing has conducted on this tool.<br>✓ This measure is very short and therefore quick to complete<br>✓ The questions focus on strengths, which is preferred for tools used with DA services	From published research:<br>✗ Some scientific testing is still needed on this tool, such as being approved for use with children, adults from ethnic, cultural, or religious backgrounds and those with accessibility needs<br>✗ This measure has not been used in a DA context	53.47	39.29	46.38
**Responder:**<br>• **Adults**	Comments from workshops:<br>✓ The Changemakers felt the questionnaire is worded positively and is easy to complete	Comments from workshops:<br>✗ Many felt the questionnaire design means responses are likely to change on a day-to-day basis and this is something to consider when voting on its usefulness in the DA-COS<br>✗ Everyone expressed that the questions are too similar and feel repetitive.<br>✗ The Changemakers felt there is little insight gained as the questions are too similar and not direct in understanding who/where your optimism comes from – e.g. from family or situations that made you feel optimistic.<br>✗ Many felt the questionnaire needed to be reworded to be trauma-informed – e.g. ‘expecting’: you may not be in the frame of mind to expect much after your experience/ expect things to go negatively			

20 Protocol Deviations

- Stages one and two were reversed due to mandatory pre-election inactivity from the UK General Election 2024.
- Due to scheduling conflicts, the research team were unable ask the VOICES advisory group to approve the survivor checklist used during the data extraction stage.
- The research team sought feedback from the Changemakers, instead of the VOICES advisory group, who approved and request no changes to the checklist.
- Initial VOICES consultation recommended the inclusion of survivor, parental, perspective. Recruited from SafeLives Pioneers, Voices at the DAC and Refuge's Survivor Panel, this supplementary group served to input a parental perspective in assessing the appropriate of included measurement tools at the consensus workshops.

21 Flowchart of OMIs identified, included, and excluded across all stages

The below PRISMA diagram depicts the flow of measurement tools across all stages. This includes the number of tools identified from the literature, the appraisal of each tool and their respective study, reflecting their accessibility within the DA-COS until the consensus stage. After the quality appraisal of OMIs (stage 2), we considered individual subscales of candidate OMIs where these captured constructs of interest. The distinction between whole measurement tools and subscales is noted below.

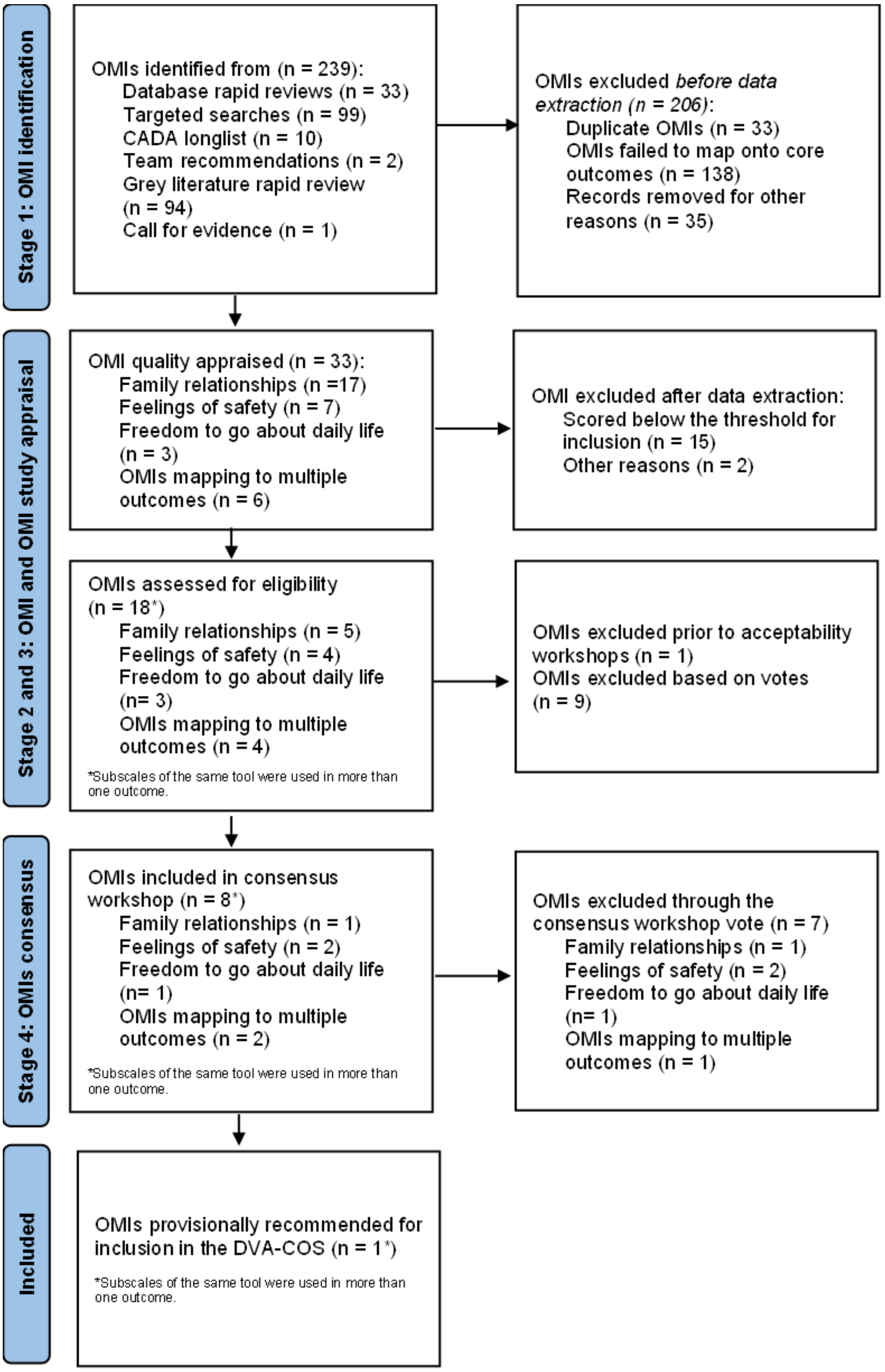

22 Core outcome definitions

The definitions of the remaining core outcomes are listed below. These definitions were informed through a series of concept workshops with domestic abuse academics, practitioners, and the Changemakers (a young people's lived-experience group affiliated with SafeLives). Comments from these workshops were thematically synthesised by the research team.

FAMILY RELATIONSHIPS: The definition of *family relationships* includes the emotional and practical aspects of a relationship. The emotional aspects can include the quality of the relationship – e.g. the emotional climate of the relationship, feelings of closeness or a 'sense of belonging'. Practical aspects include relationship functioning, such as the family script or conflict resolution. It is important to consider the relationship type within the definition of family relationships, because whoever the responder is holding in mind will influence how measurement tools are answered.

FEELINGS OF SAFETY: The definition of *feelings of safety* is context-dependent. Everyday safety can include feeling safe in different settings such as at home, in the community, or online and at different points in time. *Feelings of safety* also includes emotional/psychological safety, with the 'fear of retaliation' possibly limiting your self-expression or impacting the coping strategies you use. Relationships with family, friends, the wider community, and external agencies also affect your feelings of safety; a key consideration in this includes incorporating, where appropriate, the person/parent that harms.

FREEDOM TO GO ABOUT DAILY LIFE: The definition of *freedom to go about daily life* includes practical freedom such as financial freedom and the freedom around moving/staying in locations. This definition also includes self-freedom and its interaction with other relationships, such as having the freedom of choice and speech without fear of consequences. *Freedom to go about daily life* also includes feelings around freedom such as hope for the future or not feeling limited by your experience. Importantly, a key consideration for this core outcome is its focus on long-term outcomes.

23 Top votes comments from concept workshops

During the concept workshops, participants were invited to document their initial thoughts using the Mural whiteboard[3]. After an initial discussion, which adopted the nominal group technique (Hall et al., 2021), each participant voted on their top three comments that best captured their definition of the core outcomes. This table details the top qualitative comments voted for by participants; these informed the core outcomes definitions (supplementary material 22).

Core outcome	Changemakers	Academics	Practitioners
**Family relationships**	• Feeling like they're "walking on eggshells" around parents/siblings/ family.<br>• Impact of disclosing/discussing domestic abuse:<br>- "Your family will see you differently."<br>- "If [you] say something they will lose the 'love' from their family."<br>• Sense of belonging – feeling part of a family<br>• If in a conflict, can the parent/s handle the situation calmly?<br>• Feel they are treated fairly/equally to siblings.	• Family conflict resolution – the importance of communication and sharing.<br>• Feelings of closeness (as a family/ towards children)<br>• Grandparents as surrogate parents<br>• Who does the child see as family? Family measures ask the child who they were thinking of when completing the measure.<br>• Does the child have at least one person that they feel comfortable speaking to?<br>• How often do children talk with their family members about their feelings and concerns?	• Cultural components that construct a family – values, morals, roles, expectations, etc<br>• Importance of a child having "at least one" positive relationship with an adult<br>• The family script<br>• Identity – who am I in this space and within my family?<br>• Blame around breaking up family.<br>• Knowing more about healthy relationships and what they look like

[3] See: https://www.mural.co

	• The survivor feeling resentful or numb towards the perpetrator/s.<br>• Parent–child relationship after divorce/separation<br>• Feelings of closeness (to children or as a family)	• Time and attention – e.g. Growing Up in Scotland (GUS; national longitudinal survey) question: My family members have time for me and listen to what I have to say?<br>• What is happening within the household – e.g. household routines and regulations, such as over screen time, sleep routines, mealtime routines?	
**Feelings of safety**	• Being reassured your perpetrator is unable to access your 'safe space.'<br>• Feeling safe within yourself, not causing harm towards yourself or possible suicide risk<br>• Feeling safe need to be considered in context e.g. in the family, neighborhood, and community (in general terms) or around specific individuals (e.g. around a parent/ the individual that harm)<br>• Feeling safe forever, not just right now, tomorrow, or next week	• Feelings of safety around the parent that harms<br>• Global feelings of safety (psychological and physical)<br>• At home, school/work, and online.<br>• From child removal by social services/ court proceedings<br>• Framed as "lack of feeling unsafe"? (many survey questions might be framed this way)<br>• Did you feel safe to express your opinion/say what you think?<br>• Feelings of hypervigilance and its relation to ability to concentrate (checking safety)	• "I'm able to be my [full] self - at home/at school etc."<br>• Psychological safety: feeling settled in your own head and thoughts. What are the things that make you feel unsettled? Somatic examples etc. Promotion of words to enable the articulation of thoughts and feelings that may not previously have been articulated.<br>• Comfort in expressing feelings around safety.<br>• Psychological safety for a child: are you able to focus

	• Knowing what to do when they feel unsafe or in danger – knowing how to protect themselves in physical abuse	• Is there anywhere that you feel safe? (e.g. your room or another location)<br>• Physical safety of their own/other's property (pets and "my things")	on your homework, are you able to relax (e.g. watch TV) at home?<br>• Feelings of safety in cultural settings or in the community
**Freedom to go about daily life**	• Not feeling as though you must constantly be aware and on edge.<br>• Not feeling economically restricted or disadvantaged when with the perpetrator/s<br>• Freedom to have your own hobbies/ be able to go out with your own friends.<br>• Financial freedom<br>• Believing that every opportunity is available and not feeling disadvantaged.<br>• Not feeling as though friends, teachers, peers, other family members, etc. have been "turned against" you.<br>• If sharing something about their current DV experience, not pushing them to tell someone, or to reach for help if they do not want it but rather giving them the	• Psychological freedom to do life as you want.<br>• The luxury of not worrying – this may be more subconscious (think wellbeing): e.g. making unrestricted plans.<br>• Ability to get home safely from school/work/friends/family etc.<br>• Freedom in relation to contact (when having to arrange custody/visitation or the freedom to move locations)<br>• What does freedom look/feel like?<br>• How often do children get to choose what they do after school? Do they feel they can invite friends over to their home?<br>• Coming home and knowing things will be okay.	• "I can be myself at home/school/out and about."<br>• Opportunity to make own choices and mistakes.<br>• Consequences feel reasonable when I do something wrong.<br>• Autonomy to make own decisions.<br>• Feeling confident to get help if you need/know where to get help.<br>• Reflect personal choices (on school/ university /work) or identity (i.e. gender/sexuality)<br>• Free to identify your own identity and self-construct

	space to talk to you about it and not worry that you might tell someone.<br>• Not feeling like you have to change the way you interact with people or have to keep secrets	• Not having your life limited by a parent that harms, how this changes when things get better.<br>• Young children's freedom as expressed through ability to explore through play (e.g. sense of curiosity and fear)	

24 Thematic synthesis of concept workshop comments

This table details, for each outcome, key themes and a brief description of comments raised by participants. The table further summarises the constructs and any key considerations raised during workshops. Themes were used to inform the core outcome definitions.

Core outcome	Key themes	Summary of constructs	Key considerations
**Family relationships**	• Family script/dynamics – emotional climate, one family member dictating the tone, what is happening in the household: e.g. routines (as proxy for functioning)<br>• Sense of belonging in family – who am I within this space and my feeling, having a place and feeling wanted, happy with the relationship and structure.<br>• Feelings about family – walking on eggshells, feelings about family members, feelings towards perpetrators, anxiety/fear when with family, blame around breaking up family, anxiety will lose family love, non-abusive parent feelings.<br>• Specific to non-abusing parent – feelings of responsibility about non-abusive parent's feelings or safety and how to mitigate the impact of domestic abuse on that parent, degree of sheltering by the non-abusive parent through conflict, parent characteristics and traumas.	• Feelings of belonging and closeness in family<br>• Family emotional climate (including anxiety, fear, blame and individual feelings about different family members)<br>• Quality of family contact<br>• Family conflict resolution	• Tool needs to be flexible about who it includes: extended family, siblings, grandparents, adoptive/foster/birth, chosen.<br>• Impact of different cultural understandings of family<br>• Impact of wider context on family relationships e.g. housing, additional needs<br>• Consider impact of parenting and boundaries on family relationships, and risks of child to parent violence.<br>• Consider impact of contact with abusive parent both voluntary and court-mandated.<br>• Changemakers want agency over how and when the

	• Quality of contact with family members/extended family – being kept in mind, time and attention, parental involvement, feeling safe around family members, feelings of closeness, talking about feelings/areas of concern<br>• Family conflict resolution – role of communication, discipline, sharing, stress – feelings of tension, making mistakes, parent handling situation calmly.		person who harms is discussed, may have mixed feelings and contact can have a negative influence.
**Feelings of safety**	• Temporal, contextual, spatial safety – online, at home, at school, times of day, over time periods, where someone's safe space is.<br>• Emotional/psychological safety – worry, scared for self and others, fear of retaliation, safe within self, conflicting emotions, changing feelings and managing these, articulating feelings, feeling anxiety/ depression, somatization.<br>• Practical safety – how well you're coping, useful coping strategies, knowing what to do, what could be put in place, identifying when you feel unsafe and what to do (e.g. coping)<br>• Everyday safety – feeling safe in your daily routine, able to make mistakes at home, can you focus at home on homework, relax etc.,	• Emotional/psychological safety (including self-expression)<br>• Day-to-day safety (includes practical, everyday safety of items, safe place)<br>• Feeling safe in relationships (including impact of how safe others are)<br>• Temporal, contextual, and spatial safety<br>• Feeling safe in relation to external agencies (access to, treatment by,	• Contact with the person that harms – consider how this affects feelings of safety.<br>• Impact of community safety – consider how this affects feelings of safety.<br>• Consider how immigration/wider treatment by state affects feelings of safety.<br>• Developmental understanding of safety and how this changes over time (e.g. children and young people

	feelings of hypervigilance, and ability to concentrate.<br>• Safety of items – physical safety of property, pets, things; safety of personal information – from services or the person that harms<br>• Safety and the person that harms – knowing info etc. can’t be used against, can’t access safe space.<br>• Self-expression and feelings of safety – able to be self at home, expressing feelings/opinions<br>• Safe relationships – can you identify what a safe relationship looks like, do you have a safe relationship, family members, trusted adults/friends (overlap with family relationships)<br>• Community safety – feelings of safety in the community, societal attitudes, feeling safe in family, neighborhood, community.<br>• External agencies – awareness of support, safety in relation to external agencies, feeling supported, related to mother: e.g. immigration status, safety re: legal proceedings.	institutional safety, interference by person that harms)	relearning what safety feels like)<br>• How does someone’s identity and context affect their feelings of safety?<br>• Need to capture feelings of safety across multiple contexts and with different people

**Freedom to go about daily life**	• Money/finance related – financial freedom, not feeling pressured to work or provide finances, not feeling restricted when with the abusive parent, access to own money and freedom to buy essentials.<br>• Freedom and movement – community surveillance, moving areas, in relation to contact, choice about moving away or not, leaving the house, able to go about daily life and between settings without being accosted.<br>• Freedom in contexts – home/school/community, feel able to go to other places, retraumatizing.<br>• Online freedom – using technology without fear, free to post/share/like, use social media, not feeling monitored online.<br>• Freedom in relation to the home – coming home and knowing things will be ok, coming home doesn't make me feel bad.<br>• Practical freedom – housing stability, immigration status, accessing services, confidence to get help, human rights, safe from abusive partner using service/benefits/state, choice within this, no recourse to public funds (NRPF)<br>• Children's freedom to play – have your own hobbies, able to go out with friends, children	• Hope for the future<br>• Self-freedom (in relation to self, freedom of speech, freedom to play for children)<br>• Financial freedom<br>• Freedom in relationships (in non-abusive relationships, feelings in relationships, parenting)<br>• Freedom in different contexts (including online and at home)<br>• Freedom of movement<br>• Practical freedom	• Child age – can it be measured for children under a certain age? Until what age can non-abusive parent freedom outcome be a proxy for a child?<br>• Changemaker priorities: finance and hope for future<br>• Line between safety and freedom. Is freedom a more long-term outcome?<br>• Where school/other contexts are not safe, consider extent to which this is part of freedom and amenable to change by intervention.<br>• Consider cultural differences in understanding of freedom

choosing activities after school, feeling they can invite friends home, ability to explore through play.

- Freedom in relationships: (umbrella theme with subthemes)
    - Freedom and non-abusive relationships: able to spend time with peers, not feeling wider relationships have been turned against you, able to connect with friends/family/others, freedom to form relationships with people outside the immediate family.
    - Negative feelings: not feeling you have to change the way you react or keep secrets, not feeling as though you're walking on eggshells, not feeling constantly on edge around people,
    - Parenting behaviors: consequences feel reasonable when I do something wrong,
      do I have to ask permission to do what I want to do?
    - Hope for the future – not worrying, excitement about the future, believe have opportunities available, not having career affected by perpetrator,

	education/achievements not affected by perpetrator.<br>- Freedom of speech – freedom of expression, not afraid of speaking out, only speaking when comfortable, worry that someone might get into to trouble if you say something wrong.<br>- Freedom in relation to the self – freedom to do life as you want, freedom of own beliefs, be self, identify own identity, autonomy, make own choices, dress as want	

25 List of measurement tools included at Stage 2

The below table shows all 33 tools included within the quality appraisal stage (stage 2), which core outcome the measure maps to, and the tool's total, acceptability, and psychometric weighted scores (%), and whether the tool was shortlisted. The table does not denote individual subscales of interest.

Tool	Outcome	Weighted overall score (%)	Weighted acceptability score (%)	Weighted psychometric score (%)	Shortlisted for review
Systemic Clinical Outcome and Routine Evaluation (SCORE) Index – 15	Family relationships	60.04	39.29	80.80	No
McMaster Family Assessment Device (FAD)	Family relationships	55.05	40.48	69.62	No
Inventory of Psychosocial Functioning (IPF)	Family relationships	51.93	39.88	63.99	No
Family Adaptability and Cohesion Evaluation Scale (FACES-IV)	Family relationships	51.98	42.26	61.69	No
Beach Center Family Quality of Life Scale (With Disability)	Family relationships	70.21	54.76	85.65	Yes
Network of Relationships	Family relationships	55.19	53.87	56.51	Yes

Tool	Outcome	Weighted overall score (%)	Weighted acceptability score (%)	Weighted psychometric score (%)	Shortlisted for review
Inventory – Social Provision Scale					
Adolescent Health Review (AHR)	Family relationships	48.46	53.57	43.35	No
Multiple Indicator Cluster Survey – Family Care Indicators (MICS-FCI)	Family relationships	48.21	49.40	47.02	No
New South Wales Child Health Survey – Social Support Scale	Family relationships	48.31	46.73	49.90	No
Duke Social Support and Stress Scale (DUSOCS)	Family relationships	52.70	50.00	55.39	Yes
Brief Family Relationships Scale	Family relationships	42.34	36.90	47.77	No
Family of Origin	Family relationships	51.33	42.26	60.40	No
Child Routines Inventory	Family relationships	33.57	39.29	27.86	No
Family Routines Inventory	Family relationships	46.05	36.61	55.49	No
Social Support Rating Scale	Family relationships	0.00	0.00	0.00	No

**Tool**	**Outcome**	**Weighted overall score (%)**	**Weighted acceptability score (%)**	**Weighted psychometric score (%)**	**Shortlisted for review**
Multi-dimensional Perceived Social Support	Family relationships	72.47	65.48	79.46	Yes
Medical Outcomes Study Social Support Survey (MOS-SSS)	Family relationships	55.95	50.60	61.31	Yes
Outcomes Star – My Star	Family relationships; feelings of safety	55.37	54.46	56.27	No
CAFADA – Wellbeing and Safety	Family relationships; feelings of safety	51.43	74.11	28.75	Yes
Space for Action	Family relationships; feelings of safety; freedom to go about daily life	58.49	48.21	68.77	Yes
Integrative Hope Scale	Family relationships; freedom to go about daily life	48.61	37.50	59.72	Yes
Locus of Hope Scale	Family relationships;	43.99	37.50	50.48	Yes

**Tool**	**Outcome**	**Weighted overall score (%)**	**Weighted acceptability score (%)**	**Weighted psychometric score (%)**	**Shortlisted for review**
	freedom to go about daily life				
Comprehensive Hope Scale	Family relationships; freedom to go about daily life	21.03	1.79	40.28	No
Measure of Victim Empowerment Related to Safety (MOVERS) scale	Feelings of safety	60.36	55.95	64.77	Yes
WHO Quality of Life scale (WHOQOL-100)	Feelings of safety	58.63	48.81	68.45	Yes
Roadmap – UCLAN	Feelings of safety	48.31	64.29	32.34	Yes
Decisional Conflict Scale	Feelings of safety	72.63	60.71	84.55	Yes
Attention Bias Questionnaire	Feelings of safety	42.19	38.39	45.98	No
Brief Hypervigilance Scale	Feelings of safety	44.82	39.29	50.35	No
LGBTQ-Hypervigilance Scale	Feelings of safety	47.99	39.88	56.10	No
Urban Adolescent Hope Scale (UAHS)	Freedom to go about daily life	47.98	48.81	47.15	Yes

Tool	Outcome	Weighted overall score (%)	Weighted acceptability score (%)	Weighted psychometric score (%)	Shortlisted for review
State Optimism Measure	Freedom to go about daily life	46.38	39.29	53.47	Yes
Cognitive Processing of Trauma Scale	Freedom to go about daily life	33.13	16.07	50.20	Yes

26 List of shortlisted measurement tools for Stage 3

The below table documents the first shortlist of measurement tools to be discussed at the acceptability workshops, alongside each tool's total, acceptability, and psychometric weighted score (%). The table includes the OMIs that were recommended for inclusion as a result of the briefing workshops held with the Changemakers. The table does not denote individual subscales of interest.

	Tool	Core outcome	Weighted total score (%)	Acceptability score (%)	Psychometric score (%)
1	Beach Center Family Quality of Life Scale (With Disability)	Family relationships	70.21	54.76	85.65
2	Network of Relationships Inventory – Social Provisions Version	Family relationships	55.19	53.87	56.51
3	Medical Outcomes Study Social Support Survey (MOS-SSS)	Family relationships	55.95	50.60	61.31
4	CAFADA – Wellbeing and Safety[4]*	Family relationships and feelings of safety	51.43	74.11	28.75
5	Duke Social Support and Stress Scale (DUSOCS)	Family relationships	52.70	50.00	55.39
6	Space for Action**	Family relationships	58.49	48.21	68.77
7	Multi-dimensional Perceived Social Support	Family relationships	72.47	65.48	79.46
8	Measure of Victim Empowerment Related to Safety (MOVERS) scale	Feelings of safety	60.36	55.95	64.77
9	WHO Quality of Life scale (WHOQOL-100)	Feelings of safety	58.63	48.81	68.45

[4] The CAFADA Wellbeing and Safety tool was originally excluded from the first shortlist of 18 tools because it did not surpass the threshold for inclusion. However, when reviewed by the research team, it was noted that the measure received the highest acceptability score and received a low psychometric score; this could be attributed to the tool's extremely recent development. Despite such a low psychometric score, this tool scored 1.27% less than the shortlisted measures. To prevent the exclusion of a relevant measure, due to the recent development, this measure was presented to the Changemakers during the briefing workshop and their feedback recommended the inclusion of this tool. The measure was included as a seventh tool from this stage forward.

10	Roadmap – UCLAN	Feelings of safety	48.31	64.29	32.34
11	Decisional Conflict Scale	Feelings of safety	72.63	60.71	84.55
12	Locus of Hope Scale	Freedom to go about daily life	43.99	37.50	50.48
13	State Optimism Measure	Freedom to go about daily life	46.38	39.29	53.47
14	Cognitive Processing of Trauma Scale	Freedom to go about daily life	33.13	16.07	50.20
15	Integrative Hope Scale	Freedom to go about daily life	48.61	37.50	59.72

* Measurement tools included multiple subscales mapping to two outcomes.
** Measurement tools included subscales mapping to three outcomes.

27 List of excluded measurement tools for Stage 3

The below table documents all excluded measurement tools alongside each tool's total weighted score (%) and justification for exclusion. This table also reflects the excluded measures as recommended by the Changemakers in the briefing workshops held prior to the acceptability and feasibility workshops (stage 3). The table does not denote individual subscales of interest.

Tool	Core outcome	Weighted total score (%)	Justification
Systemic Clinical Outcome and Routine Evaluation (SCORE) Index – 15	Family relationships	60.04	Neither the overall weighted score, acceptability weighted score, nor the psychometric weighted score passed the threshold for inclusion
McMaster Family Assessment Device (FAD)	Family relationships	55.05	The acceptability weighted score did not pass the threshold for inclusion
Inventory of Psychosocial Functioning (IPF)	Family relationships	51.93	Neither the overall weighted score, acceptability weighted score, nor the psychometric weighted score passed the threshold for inclusion
Family Adaptability and Cohesion Evaluation Scale (FACES-IV)	Family relationships	51.98	The acceptability weighted score did not pass the threshold for inclusion
Outcomes Star – My Star	Feelings of safety	55.37	The questionnaire framework was not entirely relevant to the DA-COS, and the tool did not allow for specific subscales to be used. This tool heavily relied on the administrator's judgement and resulted in data collection that is necessary for inclusion in this research
Adolescent Health Review (AHR)	Family relationships	48.46	Neither the overall weighted score, acceptability weighted score, nor the psychometric weighted score passed the threshold for inclusion
Multiple Indicator Cluster Survey – Family Care Indicators (MICS-FCI)	Family relationships	48.21	Neither the overall weighted score, acceptability weighted score, nor the psychometric weighted score passed the threshold for inclusion

New South Wales Child Health Survey – Social Support Scale	Family relationships	48.31	Neither the overall weighted score, acceptability weighted score, nor the psychometric weighted score passed the threshold for inclusion
Brief Family Relationships Scale	Family relationships	42.34	Neither the overall weighted score, acceptability weighted score, nor the psychometric weighted score passed the threshold for inclusion
Family of Origin	Family relationships	51.33	Neither the overall weighted score, acceptability weighted score, nor the psychometric weighted score passed the threshold for inclusion
Child Routines Inventory	Family relationships	33.57	Neither the overall weighted score, acceptability weighted score, nor the psychometric weighted score passed the threshold for inclusion
Family Routines Inventory	Family relationships	46.05	Neither the overall weighted score, acceptability weighted score, nor the psychometric weighted score passed the threshold for inclusion
Social Support Rating Scale	Family relationships	0.00	This measurement tool was developed for the Chinese population. This was heavily used within Chinese research; however, a translated version of this measure and relevant English studies were not available
Attention Bias Questionnaire	Feelings of safety	42.19	The overall weighted score was in the bottom four for the safety outcome and the measure was therefore excluded
Brief Hypervigilance Scale	Feelings of safety	44.82	The overall weighted score was in the bottom four for the safety outcome and the measure was therefore excluded
LGBTQ-Hypervigilance Scale	Feelings of safety	47.99	The overall weighted score was in the bottom four for the safety outcome and the measure was therefore excluded
Urban Adolescent Hope Scale	Freedom to go about daily life	47.98	Although this measure did surpass the threshold of inclusion for the outcome *freedom to go about daily life*, within the data extraction stage (stage B), this measure was excluded because of comments from the Changemakers. This measure was not discussed after the briefing workshops and in the subsequent stages
Comprehensive Hope Scale	Freedom to go about daily life	21.03	Unable to access this questionnaire

28 Description of shortlisted tools

The below table describes the shortlisted tools discussed at the acceptability workshops and details all subscales of interest, the number of items within the measure/subscale of interest, and the intended reporter of the tool.

Core outcome	Tool	Description	Adult self-report	Child self-report	Adult proxy for child report
Family relationships	Multi-dimensional Perceived Social Support Scale	Full Measure<br>• 12 questions	✓	✓	
Family relationships	Beach Center Family Quality of Life Scale (With Disability)	Full Measure<br>• 25 questions<br>OR<br>Subscales of interest:<br>• Family interactions – 6 questions<br>• Parenting – 6 questions<br>• Emotional wellbeing – 4 questions		✓	✓
Family relationships	Space for Action	Subscales of interest:<br>• Community – 5 questions<br>• Friends and Family – 3 questions	✓		
Family relationships	Medical Outcome Social Support Survey (MOS-SSS)	Full measure<br>• 20 questions	✓		
Family relationships	Network of Relationships Inventory	Full measure<br>• Social Provisions Version - 39 questions	✓	✓	
Family relationships	Duke Social Support and Stress Scale (DUSOCS)	Subscale of interest:<br>• Social Support – 12 questions	✓		
Family relationships	CAFADA Wellbeing and Safety	Subscale of interest:	✓	✓	

		• Relationships Subscale – 5 questions for the child measure; 7 questions for the adult measure		
Feelings of safety	Decisional Conflict Scale	Full measure:<br>• 16 questions for traditional questionnaire<br>• 10 questions for domestic abuse adapted questionnaire	✓	
Feelings of safety	Space for Action	Subscale of interest:<br>• Wellbeing and Safety – 6 questions	✓	
Feelings of safety	World Health Organization Quality of Life 100 (WHOQOL-100)	Subscale of interest:<br>• Physical Safety and Security – 4 questions	✓	
Feelings of safety	Measure of Victim Empowerment Related to Safety (MOVERS) Scale	Full measure:<br>• 13 questions	✓	
Feelings of safety	CAFADA Wellbeing and Safety	Subscale of interest:<br>• Feeling Supported – 16 questions for adult measure and 12 questions for child measure	✓	✓
Feelings of safety	Roadmap (UCLAN)	Subscale of interest:<br>• Your Safety – 6 questions	✓	✓
Freedom to go about daily life	Space for Action	Subscales of interest:<br>• Help seeking – 3 questions.<br>• Competence – 5 questions<br>• Finances – 2 questions	✓	
Freedom to go about daily life	Integrative Hope Scale	Subscales of interest:<br>• Positive Future Orientation – 4 questions		✓

		• Lack of Perspective – 3 questions		
Freedom to go about daily life	Urban Adolescent Hope Scale	Subscale of interest:<br>• Personal Agency – 6 questions		✓
Freedom to go about daily life	State Optimism Measure	Full measure<br>• 7 questions	✓	
Freedom to go about daily life	Locus of Hope Scale	Subscale of interest:<br>• Internal Locus of Hope – 8 questions	✓	
Freedom to go about daily life	Cognitive Processing of Trauma Scale	Subscale of interest:<br>• Resolution/Acceptance – 4 questions	✓	

29 Acceptability workshop votes on final shortlisted tools progressing to the consensus workshop

The below table documents the results of the acceptability workshop votes for the final shortlist of eight tools. Each participant voted to include, exclude, or abstain from recommending an OMI to stage 4 (the consensus workshop). After the workshops, the research team summed the scores to provide an overall participant group percentage. These percentages were averaged across the participant groups to give equal weighting to each group. This appendix demonstrates the averaged percentage of ‘Yes’, ‘No’, and ‘Abstain’ votes made by domestic abuse academics, practitioners, and the Changemakers. These tools and subscales of interest continued to the consensus stage.

Core outcome	Measurement tool	Yes (%)	No (%)	Abstain (%)
Family relationships	CAFADA Wellbeing and Safety – Relationships Subscale	95	0	5
Family relationships	Medical Outcomes Study – Social Support Survey	88	4	8
Family relationships	Space for Action – Family and Friends and Community Subscales	52	25	23
Feelings of safety	CAFADA Wellbeing and Safety – Feeling Supported Subscale	93	0	7
Feelings of safety	Roadmap (UCLAN) – Your Safety Subscale	93	0	7
Feelings of safety	WHOQOL-100 – Safety Subscale	71	11	18
Freedom to go about daily life	Space for Action – Help-seeking, Competence, and Finances Subscales	60	20	20
Freedom to go about daily life	State Optimism Measure	47	33	20

30 Acceptability workshop comments on final shortlisted tools

The below table outlines the feedback the domestic abuse practitioners, academics, and Changemakers provided for the final eight shortlisted measurement tools. The comments reflect the tools' acceptability and feasibility for use within practice and as a tool for use within the DA-COS.

Measurement tool	Changemaker comments	Practitioner comments	Researcher comments
CAFADA Wellbeing and Safety – Relationships Subscale	Pros:<br>• Really liked the statements.<br>• Easy to understand.<br>• Goes into the correct level of detail.<br>Cons:<br>• References only "mum" – should allow space for more people.<br>• Preference for a four-point Likert scale and a numbered response scale<br>• Child version needs developing – some young people preferred answering the adult scale (14+)	Pros:<br>• Directly relevant and captures what is focused on during interventions.<br>• Free text box – has been requested by other services for other measures designed for young people.<br>• Open, accessible, and feels warm.<br>• Liked the differentiation between adult and child versions.<br>• Language is simple and clear.<br>Cons:<br>• Would like to add items exploring relationships with agency workers.<br>• Implications around gendered language	Pros:<br>• Free text box is well liked as this can capture the journey of change – suggestion to include a voice recording option.<br>• Tool is considered child and adult friendly.<br>• Covers a breadth of topics (compared to the other measures discussed)<br>• Co-developed with survivors<br>• Would be easy to implement into practice.<br>• Looks brief.<br>• Empowering for those completing the survey<br>Cons:<br>• Adult measure overlaps with feelings of safety – unsure if the child measure does as well?

Medical Outcomes Study – Social Support Survey	Pros:<br>• Liked specific questions – 15, 16, 19, and 20.<br>• Some preferred to have more questions as the questions were detailed.<br>• Questions were less subjective, so they would be easier to answer.<br>Cons:<br>• Use of the term "someone" gives a repetitive feel.<br>• Difficulty quantifying the different response scale points – "a little of the time" vs "some of the time"; preference for an agree–disagree response scale.<br>• Some felt the questionnaire was too lengthy.<br>• Disliked the format	Pros:<br>• The use of "someone" is vague and could refer to any trusted person.<br>• Liked the language used.<br>• Tool could be used by UKTC or with services such as Refuge.<br>• It feels relevant and covers a lot of different areas that are relatable regarding family relationships and day-to-day living/needs.<br>• It gives a good indicator for what someone's life looks like<br>Cons:<br>• Tool may not be appropriate for domestic abuse settings – more appropriate for social care	Pros:<br>• Liked that the statements could apply to any form of social support not just family – e.g. if you've been estranged from your family.<br>• Question 3 is an important question 'someone you can count on to listen to you when you need to talk.'<br>• Clear wording<br>• Questions are better at getting more detailed information compared to other tools – e.g. 'getting to the heart of what you want to measure.'<br>• Intuitive<br>• Behaviors within the statements capture what you can actually gain from different relationships e.g. friends are likely to spend time with you, romantic partner likely to give you hugs etc.<br>Cons:<br>• Friendships are not discussed in the current tool but are an important support system to include.<br>• While the definition of family is broad, unsure if this appropriately

			captures the person that harm's perspective.<br>• Too long<br>• Unsure if this measure has been developed using trauma informed principles, the tool currently could be considered upsetting/triggering.<br>• Questionnaire could be interpreted differently based on circumstantial factors e.g. time of year.<br>• Young people, because of social desirability, could disengage from this tool – could be perceived as judgmental?
Space for Action – Family and Friends and Community Subscales	Pros:<br>• No pro comments were raised.<br>Cons:<br>• Disliked the past tense and timeframe.<br>• Disliked the response scale-preference for a four-point Likert scale and an "I don't know option."	Pros:<br>• Liked the topics but requires a lot of adaptation.<br>Cons:<br>• Subjective and retrospective<br>• Disliked past tense wording	Pros:<br>• Broadly relevant to the outcome<br>• Liked the community/ education questions.<br>Cons:<br>• Too few questions on family relationships<br>• Too open to interpretation – e.g. "enough"<br>• Tool is useful in understanding coercive control and relationships

	• Friend and family subscale is subjective – e.g. "enough."<br>• More questions are needed to explore family relationships.<br>• Too focused on the community		but not appropriate for rebuilding relationships.<br>• Disliked the past tense wording.<br>• Does include the person that harm's perspective, however there is not enough exploration.<br>• Unclear of the timeframe the tool should be used with<br>• Friends and family questions are not meaningful – you want to know they have someone they rely on rather than the number of relationships.
CAFADA Wellbeing and Safety – Feeling Supported Subscale	Pros:<br>• Simple<br>• Free text box – some questions need to be expanded.<br>• Attractive tool<br>• Scale is simple to implement.<br>• Easy to understand the questions and how they map to feelings of safety.<br>Cons:<br>• Disliked the neutral/not sure how to answer options.	Pros:<br>• Highly relevant to the feelings of safety<br>• Accessible and inclusive<br>• Simple with clear language<br>Cons:<br>• Question "I feel safe at home" may not be relevant for all families so should be adapted – e.g. the Traveler community or those living in refuges.	Pros:<br>• Highly relevant – asks about different types of safety.<br>• Free text box for clarification<br>• Both an adult and child version<br>• Clear this tool was developed for use in DA contexts.<br>• Clear meaning and interpretability – intuitive<br>• Liked that this scored so well on acceptability (at the data extraction stage)<br>Cons:

	• Too focused on "mum" Some items are confusing – e.g. I feel my mum and family are safe from domestic abuse and hurt in my family.'<br>• Some items in the adult version are more appropriate for older children/young people.		• Some items in the child version appear to be relevant.<br>• No reference to online safety<br>• Slightly too long but preferred as this feels more well-rounded when capturing this outcome
Roadmap (UCLAN) – Your Safety Subscale	Pros:<br>• Tool is succinct.<br>• Covers a lot of important topics.<br>• Liked that the questions were framed within "the past 2 weeks" as this gives a clear understanding of safety on a day-to-day basis.<br>Cons:<br>• Disliked the use of past tense.<br>• Preferred a four-point Likert scale.	Pros:<br>• First questionnaire to include a question about online safety.<br>Cons:<br>• Question "I feel safe and secure" could be problematic as if their home was safe there would be no motivation to leave.<br>• Gendered language should be changed.<br>• Many adaptations are needed	Pros:<br>• Short but distinct dimensions of safety are explored.<br>• Clear and immediate timeframe – useful from an administration and data perspective<br>• Includes question around children and contact with person that harms (disliked that this was gendered)<br>• Feels appropriate for survivors.<br>• Questions are generic with an appropriate level of vagueness to be accessible to many.<br>Cons:<br>• No free text box

	• Prefer the addition of a free text box		• The nature of abuse (on-off patterns) means that the 2-week timeframe may be inappropriate for capturing someone's experience.<br>• Unsure around how questions around contact will impact feelings of safety – is this tool enough to capture this complexity?
WHOQOL-100 – Safety Subscale	Pros:<br>• Simple and easy for administrator to know if someone is feeling safe or not.<br>Cons:<br>• Question 2 is subjective – may lack the awareness that your environment is unsafe if it's the responder's norm.<br>• Specific examples of what safe and secure mean are needed.<br>• Disliked the different Likert scales and that this was not made clearer.<br>• Dislikes the neutral response option	Pros:<br>• Tool is culturally sensitive/ culturally competent.<br>• Could use this tool with a child and this would be useful at any stage of intervention.<br>• Simple and short while covering the main topics of feeling safe.<br>Cons:<br>• Some language is inaccessible.<br>• Measure needs to be expanded	Pros:<br>• Liked the use of the term "worry."<br>• Simple in distinguishing feelings vs physical safety<br>Cons:<br>• Some items are irrelevant – e.g. community violence or physical safety questions.<br>• Very short<br>• Open to interpretation and this will impact how service users are likely to respond e.g. which environment is being held in mind?<br>• Feels reductionist.<br>• Preference for "daily life" rather than asking about living in safe and secure environments (commentary from researcher who discussed this

			tool with survivors within their research)
Space for Action – Help-seeking, Competence, and Finances Subscales	Pros:<br>• Liked the inclusion of finances and budgeting – reflects different types of abuse.<br>• One Changemaker felt this tool "captured everything that they asked for."<br>• First-person questions feel more personal and like the responder is involved (less like an assessment)<br><br>Cons:<br>• Not relevant for children – needs adapting.<br>• Past tense questions are disliked.<br>• Disliked the Likert scale – preference for four points	Pros:<br>• Liked the help-seeking subscale; felt most relevant to this outcome.<br>Cons:<br>• Disliked the past tense questions.<br>• Focus on finances not applicable to children.<br>• Irrelevant questions – e.g. confidence with dealing with authorities<br>• Term "competence" is loaded	Pros:<br>• Straightforward and deals with concrete concepts<br>• Some items are highly relevant.<br>• Simple tool<br>• Appropriate for use in domestic abuse settings<br>Cons:<br>• Past tense questions are off-putting.<br>• Unsure if the competence subscale is appropriate for the outcome – reflects for coping rather than something you could aspire towards
State Optimism Measure	Pros:<br>• Liked the use of a timeframe (only in question 4) – this style of question is easier to answer.	Pros:<br>• Liked that the measure focuses on the positives.<br>• Hope for the future is important to capture.<br>Cons:	Pros:<br>• Brief and concise<br>• Easy to administer and complete.<br>Cons:<br>• Too general

	• Positively worded and refers to current feelings.<br>• Like the length – short<br>• Feels balanced.<br>Cons:<br>• Disliked the scale – preference for four-point Likert scale.<br>• Some items are too far removed to capture freedom to go about daily life.<br>• Free text box<br>• Doesn't capture where hope comes from – e.g. internal hope or family/people around you.<br>Indirect reflection of outcome so unlikely to flag issues for children and young people	• Many items are captured in other measures included in DA-COS (WEMWBS)<br>• Not relevant to the core outcome<br>• Items are too similar and will be difficult for young people to understand the differences.	• Preference for this tool to be used with an excluded measure (Integrative Hope Scale) to understand current and future feelings around freedom.<br>• Feels repetitive

31 Acceptability workshop votes for excluded tools

The below table documents the results of the acceptability workshop votes for the excluded tools and subscales of interest; this documents the percentage of 'Yes', 'No', and 'Abstain' votes made by domestic abuse academics, practitioners, and the Changemakers.

Core outcome	Measurement tool	Yes (%)	No (%)	Abstain (%)
Family relationships	Multi-dimensional Perceived Social Support	41	13	45
Family relationships	Beach Center Family Quality of Life Scale (With Disability)	7	62	31
Family relationships	Network of Relationships Inventory – Social Provisions Version	25	62	13
Family relationships	Duke Social Support and Stress Scale (DUSOCS)	18	64	18
Feelings of safety	Decisional Conflict Scale	12	52	36
Feelings of safety	Measure of Victim Empowerment Related to Safety (MOVERS) scale	0	37	63
Feelings of safety	Space For Action – Wellbeing and Safety Subscale	60	27	13
Freedom to go about daily life	Integrative Hope Scale	30	27	43
Freedom to go about daily life	Urban Adolescent Hope Scale*	N/A	100	N/A
Freedom to go about daily life	Locus of Hope Scale	25	48	27
Freedom to go about daily life	Cognitive Processing of Trauma Scale	0	93	7

* In line with the study's commitment to prioritise survivor feedback, the Urban Adolescent Hope Scale was excluded from voting for practitioner and academic workshops. This decision to exclude this measure resulted on the Changemakers' strong opposition to including this tool and their recommendations to exclude.

32 Acceptability workshop comments on excluded tools

The below table outlines the feedback the domestic abuse practitioners, academics, and Changemakers provided for the excluded measurement tools/subscales of interest. Similar to supplementary material 22, the comments below reflect the tools' acceptability and feasibility for use within practice and as a tool for use within the DA-COS.

Measurement tool	Changemaker comments	Practitioner comments	Researcher comments
Multi-dimensional Perceived Social Support	Pros:<br>• Preference for the response scale to have no neutral option.<br>• Statements are clear and concise – not overwhelming.<br>• Tool is simple and quick to complete.<br>• Talks about family relationships well.<br>• Liked that the items were in first person.<br><br>Cons:<br>• Confusion around the phrase "special person"<br>• Difficulty about response scale – how to quantify "strongly agree"	Pros:<br>• Positive statements<br>• "Special person" is preferred as the individual is not pre-defined.<br>• Allows subjectivity and inclusivity – tool is relatable to most people's circumstances.<br>• Tool could be used to inform risk assessments/safety planning.<br><br>Cons:<br>• Phrase "my family" used repeatedly and this could be triggering – e.g. care-experienced children.<br>• 11-item tool is too long for children	Pros:<br>• "Special person" is useful because estranged from family and friends.<br>• Likes items asking about friends – strong evidence about the support of friends; however, could be triggering for those isolated.<br>• Wording is warm and friendly.<br>• Degree of flexibility/adaptability for different ages and stages<br>• Measure isn't too long.<br><br>Cons:<br>• Many items are not relevant to the family relationships core outcome.

		• Items are too similar and the nuance between items are not accessible to children.<br>• Limited specification to domestic abuse	• Response scale is somewhat leading and may not need to include seven points.<br>• No reference to the person that harms<br>• Could feel overwhelming.<br>• Tool may not be accessible to care-experienced children.
Beach Center Family Quality of Life Scale (With Disability)	Pros:<br>• Liked that the items were worded in first person as it made the tool feel personal.<br><br>Cons:<br>• Formatting looks busier/ clunky.<br>• Questions, especially starting with "my family", are repetitive and boring.<br>• Language is not simple or friendly.<br>• Too wordy<br>• Many items felt irrelevant as it references family in general.<br>• Disliked the satisfaction rating scale.	Pros:<br>• Comprehensive – covers many important areas.<br>• Good tool for initial assessment – gaining lots of information about family.<br>• Preference for measuring satisfaction.<br><br>Cons:<br>• Very long – unfeasible to complete in practice.<br>• Some questions are irrelevant – recommended only six items.<br>• Reads very clinical/as an assessment.<br>• Some items – e.g. "my family enjoys spending time together"	Pros:<br>• Some topics are important to open up discussions.<br>• Some of the practical questions are highly relevant – e.g. medical care, transport, or trusting an agency.<br>• Measures satisfaction<br>• Strengths-based statements<br><br>Cons:<br>• Too long – preference to select a few items (disliked the subscale)<br>• Captures quality of life, which can be useful but not enough focus on family relationships outcome.

	• Many felt the items were not applicable/accessible to them	– inappropriate for domestic abuse contexts.	• Questions are vague/generic and oddly worded (not intuitive) – likely to be difficult to self-complete.<br>• Question wording problematic and potentially influenced by social desirability biases.<br>• Doesn't distinguish between the non-harmful parent and parent that harms.<br>• For the statement "satisfied that family enjoys spending time together", whose perspective are you considering when answering?<br>• Statements read similar to case management tools – assessment focused.<br>• Some statements could be viewed as minimizing – e.g. "life's ups and downs."
Network of Relationships Inventory – Social Provisions Version	Pros:<br>• Liked that you can define your relationships and then don't have to think about who you are holding in mind.	Pros:<br>• Helps the child pinpoint supportive/ less supportive relationships.<br>• Accessible	Pros:<br>• Does allow for the person that harms (mother or father figures) to be discussed.

	• Really useful for older children/adolescents, not likely suitable for younger children<br>• Scale is clear and concise.<br><br>Cons:<br>• Doesn't accommodate for those that don't have a specific relationship (e.g. if you don't have grandparents/ same-sex friends there is no option to state that)<br>• Too long and can be overwhelming.<br>• Repetitive in terms of format and some of the questions<br>• Response format is complicated.<br>• Changemakers asked if it's possible to answer one relationship at a time?	Cons:<br>• Very long – unfeasible to complete in practice.<br>• Defining the relationships may be challenging	• Thorough when exploring relationships.<br>• Liked that responder can outline who they are holding in mind.<br><br>Cons:<br>• Complex<br>• Too long – difficult to implement, especially with DA-COS<br>• Many items could be problematic – e.g. fighting, secrets, and conflict items are worded in a minimizing way.<br>• Perspectives on the person that harms should be included in a better way.<br>• Need to be motivated to complete this questionnaire.<br>• Preference for this measure to be administrator-led, not self-reported.<br>• Some items are irrelevant – e.g. exploring time spent together – or not applicable to

			all relationships (whether relationship will last)<br>• Potential for misunderstanding – with items e.g. conflict doesn't explore culture or context or with the whole measure, are you comparing relationships?
Duke Social Support and Stress Scale (DUSOCS)	Pros:<br>• Short and concise<br>• Can explore supportiveness through the designated relationships.<br>• Don't have to over-analyze.<br>• Defines "supportive" (however, this is a limited definition)<br><br>Cons:<br>• Difficulty distinguishing "no support" from "no such person" category.<br>• Not detailed enough/too broad<br>• Reference to church only is not inclusive.<br>• Feels like you are "ticking on a list."	Pros:<br>• No pros discussed<br><br>Cons:<br>• Church item is not inclusive.<br>• Doesn't capture the focus of interventions/measure change.<br>• Outdate implications around faith and employment items	Pros:<br>• Liked that relationships beyond nuclear family are explored.<br>• Can specify no relationship without providing an explanation; therefore, scope to explore more difficult aspects of family relationships.<br>• Attractive tool<br>• Good length questionnaire<br><br>Cons:<br>• Specific definition of family relationships<br>• "How supportive" is subjective and vague.<br>• Feels like a precursor tool.

	• Some categories need separating – e.g. parents or siblings.<br>• Difficult to answer as some relationships don't apply		• Possible confusion between the "no such person" and "no support" category<br>• Difficulty answering sibling question if you have multiple siblings.<br>• Tool feels old-fashioned.
Decisional Conflict Scale	Pros:<br>• DA-adapted measure was preferred but felt repetitive.<br><br>Cons:<br>• Questions are too general.<br>• Disliked formatting and response scale<br>• Repetitive use of "I am."<br>• Question "do you know the risk of staying in the relationship" is inappropriate/judgmental.<br>• Some items are not intuitive and require a lot of thought to answer.	Pros:<br>• Clear and focused – complementary to other questionnaires if used in collaboration.<br><br>Cons:<br>• Not child friendly<br>• Some questions are irrelevant.<br>• Unsure how the practitioner would understand if the child felt safe.	Pros:<br>• Adapted tool is more relevant to core outcome.<br>• Liked questions referring to support and advice without pressure.<br>• Some items in the original measure could capture freedom to go about daily life.<br><br>Cons:<br>• Original tool is too far removed from aims of outcome.<br>• Original measure is too long.<br>• Victim blaming/judgmental language, especially placed on the non-harmful parent – e.g.

			"keeping yourself and your children safe."<br>• The original tool's focus on choice is not appropriate for domestic abuse – survivors don't view their experience as a choice to leave or stay.<br>• Original measure needs adapting and adapted measure needs validating.<br>• Adapted measure could be considered patronizing.<br>• First six questions feel too simplistic and are off-putting.
Measure of Victim Empowerment Related to Safety (MOVERS) scale	Pros:<br>• Liked the level of detail in the questions.<br><br>Cons:<br>• Disliked question 1<br>• Difficult to quantify the scale – e.g. "half of the time."<br>• Question "I have to give up too much to keep safe" was strongly disliked.<br>• Some questions feel repetitive.	Pros:<br>• With adaptations this could be accessible for older children<br><br>Cons:<br>• Tool is not accessible for children – difficult for them to understand the nuance of the questions.<br>• For self-report, the focus of decision making would be on the child, but they may not	Pros:<br>• Was clearly relevant to the core outcome.<br>• Some good statements – e.g. comfortable asking for help to keep safe.<br><br>Cons:<br>• Too many questions – feels overwhelming.<br>• Language is clunky and not user friendly.

	• Question wording is confusing and not direct, so is difficult to understand and answer.	have that power to make decisions, which could translate to feelings of pressure.<br>• Too intellectualized	• Not easy to understand.<br>• Doesn't clarify safety first.<br>• Too great onus on non-harmful parent<br>• Unsure how the tool would cope if the non-harmful parent/child was still in the abusive situation.<br>• Safety is relative and this nuance is not captured – e.g. not engaging/ challenging to remain safe.<br>• Reads like a service evaluation form.<br>• Explores safety in one way – family and individual but they should be separated.<br>• Some items are ambiguous.<br>• Disliked references to goals
Space For Action – Wellbeing and Safety Subscale	Pros:<br>• Simple and short – not overwhelming<br><br>Cons:<br>• Doesn't consider how safe you are around certain individuals.	Pros:<br>• Questions are simple and easy to understand.<br>• Questions about home could be used to understand family relationships/dynamics.	Pros:<br>• Generally easy to use and simple.<br>• First three items are highly relevant.<br>• Like questions such as choice around dressing

	• Disliked statements are in past tense.<br>• Question "I like my house": there is the assumption this is about safety and not something non-domestic abuse-related – e.g. disliking your room.	• Retrospective so could be used to measure different timepoints.<br><br>Cons:<br>• If moved, question "I liked the house I lived in" is really inappropriate.<br>• Age is an important factor – teenagers are likely to dislike their environment, but this is not domestic abuse-related.<br>• Not relevant to the outcome<br>• Not trauma-informed<br>• Question "my home was safe" is not likely to change with intervention.	• Good number of questions<br><br>Cons:<br>• Missing topics: safety within relationships, practical safety, psychological safety, or online safety<br>• Items are more precise than about general feelings.<br>• Some items could map to freedom to go about daily life.
Integrative Hope Scale	Pros:<br>• Liked reference to future hope.<br>• Questionnaire maps to outcome<br><br>Cons:<br>• Phrasing needs adapting – e.g. "pinned down/bothered by troubles."	Pros:<br>• Positive-coded subscales will be more well received.<br><br>Cons:<br>• Negative-coded items could be difficult for a child to understand.	Pros:<br>• Some items are highly relevant.<br>• In practice the questionnaire could be useful in helping service user communicate and then the practitioner provides more appropriate action plans around any concerning items

	• A lot of negative statements made the tool feel “attacking” in nature	• Many felt hope doesn’t map to freedom – reference that autonomy as a construct more appropriately maps to freedom to go about daily life compared to the construct hope.<br>• Feels emotive and subjective.<br>• Not for a therapeutic space	• Positive future orientation subscales are strengths-based.<br><br>Cons:<br>• Not specific enough so open to interpretation<br>• A bit too broad<br>• Could be upsetting if completed alone.<br>• Liked the mix of positive and negative framed questions but there should be a better mix: negative items should be framed around current experiences whereas positive items focus on the future.
Urban Adolescent Hope Scale	Pros:<br>• No pros discussed<br><br>Cons:<br>• Disliked this tool – questions do not get us to know what we want to find out.<br>• Start of the week might have positive outlook but might not at the end of the week.	• Measure excluded during briefing workshop with Changemakers; no other participants discussed or voted on this tool	

	• Too broad – free text would be helpful but might put people off filing it in<br>• Free text box: for subjective statements – e.g. "I have goals" – does not need a text box.<br>• "I have goals" is broad – this should be more specific: e.g. future goals or career options.		
Locus of Hope Scale	Pros:<br>• Liked the four-point Likert scale.<br>• Straight to the point<br>• Liked the positive focus.<br><br>Cons:<br>• Unsure this is appropriate for all ages.<br>• Disliked the wording of certain questions – e.g. "I energetically pursue my goals"; "I can personally get around any problem."<br>• Likely to feel deflated if scoring low on this tool.	Pros:<br>• No pros discussed<br><br>Cons:<br>• Disliked the focus on success and goals – should focus on what the goals are rather than whether they are met.<br>• Inappropriate language of some items – e.g. "past experience prepared me for the future."<br>• Items around faith should not be used.<br>• Not accessible	Pros:<br>• For some responders, goals can be really positive.<br><br>Cons:<br>• Inappropriate for outcomes – sounds like a career reflection survey.<br>• Disliked the lack of a neutral option in the response scale.<br>• Disliked the wording of the items.

	• Statements are confusing and open to interpretation so will not reflect responder's true feelings.		
Cognitive Processing of Trauma Scale	Pros:<br>• No pros discussed<br><br>Cons:<br>• Especially disliked questions 4 and 5<br>• Questions are open to interpretation.<br>• Doesn't capture the outcome.<br>• Difficulty understanding the response scale – what's the difference between "slightly" and "moderately"?<br>• You may never come to terms with your experience, so you are unlikely to get what you need from this tool	Pros:<br>• No pros discussed<br><br>Cons:<br>• Not relevant to the outcome<br>• Inappropriate to expect a child to move past "the event."	Pros:<br>• Recognizes the trauma of domestic abuse.<br><br>Cons:<br>• Appropriate as a post-trauma questionnaire but irrelevant to outcome<br>• Measure feels limited.

33 Survivor feedback workshop summary

The below table documents the comments made by SafeLives Changemakers and Pioneers as part of the survivor feedback workshops. These were scheduled for the survivor participants who were unable to attend the consensus workshop to share their perspectives on the final eight measurement tools. The Changemaker comments were shared within the pre-workshop pack, and the Pioneer comments were used as wider discussion points during the consensus workshop.

<table>
<tr><th>Measurement tool</th><th>Changemaker feedback</th><th>Pioneer feedback</th></tr>
<tr><td>1A: CAFADA Wellbeing and Safety – Relationships Subscale</td><td>Pros:
This measure was generally well liked by the Changemakers:
- The correct amount of detail
- Easy to understand
Liked that the statements mentioned specific family members
Liked that there was a free text box to expand on answers
Cons:
Child version of this tool could be improved
- Gendered language of “mum” could be changed to allow space for others to be considered
- Some of the older children may find the child version inaccessible/prefer to answer the adult version
Preference for four-point answer and the number response
Wording of questions needed to be changed
- Factoring different family structures – e.g. families without a mother/mother figure, or families with multiple children
- Vagueness – e.g. “difficult time” or “look out for me” could vary in meaning
- For the question “mum knows important things about me” the items in the brackets could be limiting</td><td>Be mindful of the impact of questions – depending on where someone is in their journey the questions could be problematic
Cultural awareness
Items needed to be worded more sensitively – wording reflects mother’s responsibility or can be presumptive
Who is regarded as family – culturally some families include extended members; who is the child regarding?</td></tr>
</table>

	• Formatting: white text, colours, and font/font size may need to be adjusted<br>- (Young Person's Liaison) Practitioners will be printing questionnaires in black and white as this is cost-effective and in this format the scales will use a lot of black	
1B: Medical Outcomes Study – Social Support Survey	Pros<br>• This tool was liked, especially the following questions: Q15, 16, 19, and 20<br>• Liked the questions 1–4, with a preference to change them to use an agree/disagree format<br>- Liked the frequency answering scale for other questions<br>• Questions were specific and detailed<br>- Some were happy to have more questions that provided enough detail about family relationships rather than less questions<br>- The more concise the question is, the harder it is to answer<br>• The wording of questions is less subjective and therefore easier to answer<br>• Liked that the questions explored different relationships<br>• Liked that the questionnaire was accessible<br><br>Cons:<br>• Use of the term "someone" made the tool feel too repetitive<br>• Some will find it hard to differentiate between the answering options "a little of the time" and "some of the time"<br>- Suggestion to change the answer format to agree/disagree styles – this suggestion was changed to reword responses to "regularly" and "occasionally"<br>• The questionnaire looks cramped	• Mindful of impact – what's the fallout if someone answers no?<br>• Some questions are unclear

	- Could be the font/formatting – this was reiterated in the survivor feedback workshop as the formatting could make the tool inaccessible<br>- Some felt there were too many questions; however, not all agreed<br>• Question 1 does not tell you much without context and might not be appropriate for young people who are close with different groups of people<br>- Suggestion to reword: "how many people do you feel close to/are in your inner circle?"	
1C: Space for Action Scale – Family & Friends; Communities Subscales	Pros:<br>• Liked that some of the statements are specific and help you [researcher] gain more information.<br>Cons:<br>• Disliked that the questions are written in the past tense.<br>- Suggestion to add a timeframe to frame the statements – e.g. the past three months.<br>• Wanted to reduce the Likert Scale from seven points to four to remove the neutral options.<br>- Suggestion to include an "I don't want to answer" option.<br>• Questions were minimal and need developing:<br>- The term "enough" is subjective and difficult to answer – reiterated in survivor feedback workshop but could be reworded to "I am comfortable with the number of friends I have."<br>- More focus on friends and family and less on the community – e.g. for young people you could ask which friends you go to if you are worried or having fun with to outline who you speak to for emotional support/advice.	• Impact in different contexts – honor-based violence (HBV)

	• If implementing a timeframe to answer questions, this might be difficult to see changes when asking about communities as this is very dependent on people's circumstances.<br>• Communities: people may be a part of different communities that serve different purposes and therefore responses may differ depending on who you keep in mind<br>• Wording may not be accessible to young people – "I don't know what communities I am in" or "what does an 'active member' mean?"	
2A: CAFADA Wellbeing and Safety – Feeling Support Subscale	Pros:<br>• This questionnaire was simple and easy to understand.<br>• Liked the free text box as there are some questions that will need to be expanded upon<br>• The formatting of this questionnaire is well liked.<br>• The answering format is simple.<br>• There are a lot of questions, and this helps to paint a picture.<br><br>Cons:<br>• The neutral/not sure option was not liked.<br>• Did not like the gendered language – "mum."<br>• Some questions were confusing – e.g. "I feel my mum and family are safe from domestic abuse and hurt in my family."<br>- For the question "I have the things I need" the scoring "not a lot" to "a lot" does not make sense – suggested to use a frequency scale: e.g. rarely.<br>- Similar change was suggested for the question "I feel safe at home" on the child measure	• Prefer the items include a range of examples to be more inclusive – e.g. the important of religious schools

2B: Roadmap (UCLAN) – Your Safety Subscale	Pros:<br>• The measure is short but covers a lot of relevant topics.<br>• Liked that a timeframe was provided as this allows people to get across how safe they feel on a day-to-day basis – maybe this could be longer if used in the DA-COS (e.g. three months)<br>• Format is neat.<br><br>Cons:<br>• It was disliked that the statements were written in the past tense.<br>• Would prefer to have a four-point Likert scale.<br>• It was suggested to add a free text box to expand on answers/outline why people answered in the way they had.<br>- One suggestion alongside the above is that asking, "what does safe look like to you?" as a free text box could provide a benchmark for practitioners to work from<br>- Free text box when asking about online and neighborhood is important as these topics are very broad – e.g. might be safe on TikTok but not Google as this is monitored.<br>• For the question "I have felt that it is safe for my children to spend time with their father (if relevant)", this should be changed to reflect that the "father" may not always be the perpetrator.<br>• To improve the questionnaire there should be a question like "It is safe to express my views and opinions."	• No additional comments – points already raised by Changemakers
2C: WHOQOL-100 – Safety Subscale	Pros:<br>• The questions were simple and easy to understand whether someone feels safe or not.	• Measure was specific and person-centered

	• Some liked that the Likert statements were not the same, but the formatting should make this clearer<br>- Others felt that this made the questionnaire disjointed.<br><br>Cons:<br>• The neutral answering option was disliked.<br>• Specific to question 2, clarity is needed as someone may think their environment is safe as that's their norm rather than their environment being objectively safe.<br>- It was suggested that specific examples of what might be safe and secure are needed.<br>• With only four questions this questionnaire feels generic and does not give you [researcher] much insight<br>- Additional information is needed.<br>• Some questions can be misinterpreted.<br>• Question 3 needs to be separated into two questions – someone may feel secure but unsafe.<br>• The questionnaire should be formatted into a table.	
3A: Space for Action – Help-seeking; Competence; Finance Subscales	Pros:<br>• It was liked that questions focused on finances and budgets because it reflects the different types of abuse and is what should be focused on<br>- In the survivor feedback workshop, this was changed with a preference for the help-seeking subscale as the most relevant to DA contexts.<br>• The personal wording of the statements "I" or "me" allows the responder to feel more involved and this wording was preferred.	• Cultural awareness – in some cultures it is not common to speak about feelings, and they may not have the capacity or have access to the language to speak about

	• The number of questions does not feel too long.<br>Cons:<br>• The statements need to be changed to present tense.<br>• It was preferred that the Likert scale be reduced from seven to four points.<br>• The questionnaire feels like it focuses on the "surface level."<br>• The term "comfortable" is a bit too ambiguous, especially when in reference to income.<br>• Questions about managing incomes/budgets or dealing with authorities are not likely to be relevant to children and young people.	their feelings if they cannot access English
3B: State Optimism Measure	Pros:<br>• Liked that the statements are time-specific as this makes them easier to answer – e.g. question 4.<br>• The positive wording was liked.<br>• Liked that the measure measured current feelings.<br>• Some liked the answering format.<br>• The measure is a good length and not too much strain on the person.<br>• This measure is balanced.<br>Cons:<br>• Some of the questions were not entirely relevant or are indirect and so might not flag issues – there is little depth to the questions.<br>• The questions do not outline where the hope comes from – e.g. from family or people around you?<br>• It was suggested for a free text box to be included – e.g. if someone answers question 7 as "strongly disagree", then it would be important to know more:	• Language may be difficult to access.<br>• Addition of a free text box has been raised previously.

	- Suggestion to include a new system to gain more information – e.g. if you choose 1, 2, or 3 then please explain why.<br>• The instruction is over the top – could just say "currently."<br>• Format means it can be confusing about which question you are answering – preference for a tick box or circle method for answering the questionnaire.<br>• Stagger the introduction of measures – e.g. questions should first reference safety and relationships and then reference how optimistic you are

34 Comments for the provisionally recommended measurement tools

This table summarises the feedback on the recommended measurement tools made by attendees of the consensus workshop.

Measurement tool	Positive comments	Areas for improvement/adaptations required
1A: CAFADA Wellbeing and Safety – Relationships Subscale	• Good mechanism for unpicking detail and helping to meaningfully understand someone's journey.<br>• Helpful language without jargon<br>• *Feels* right and better from a lived-experience perspective, easy to process, especially if you're in a place of trauma.<br>• Provides a good focus on family relationships.<br>• Questions are simple and accessible.<br>• Free text box<br>• Feels balanced between the non-harmful parent and the child (complementary)<br>• Preferred length (compared with Tool 1B) so less traumatizing/stressful to complete, especially for children.<br>• Visually appealing<br>• The adult measure feels more complete and requires fewer changes.<br>• Easy to train administrators to implement this tool.<br>• Co-developed with survivors	• Definitions are missing, including family or main caregiver.<br>• Not applicable in all contexts – e.g. doesn't consider families with babies or unborn babies.<br>• Needs to include communities – e.g. faith communities.<br>• Statements are totalizing; therefore, the tool feels appropriate at the final stages of dealing with the experience but not for capturing the journey/process.<br>• Doesn't appear to capture the dynamic nature of domestic abuse.<br>• Asking whether the parent has spoken to the child about domestic abuse can be problematic and might be used against them (parental alienation) – this question needs an "I'd rather not say" option.<br>• Needs adapting and expanding for children of different ages, with the suggestion of two measures (one for older children and another for younger children)

		• Gendered language – assumes “mum” is the non-harmful parent.<br>• Focus on “mum” is not encompassing of different family structures.<br>• Unsure how non-verbal children will use this measure.<br>• Unsure if the free text box is intended to be used in analysis.<br>• Missing certain topics – e.g. conflict resolution<br>• Doesn’t focus on emotions (relative to tool 1B)<br>• Doesn’t explore behaviors a parent notices in their child without the child disclosing.<br>• Some items are ambiguous – e.g. “being treated fairly”, “my family look out for me”, or “feeling supported as a parent.”
2A: CAFADA Wellbeing and Safety – Feeling Support Subscale	• Standardized approach is helpful to practitioners.<br>• Tool is service orientated.<br>• Clear/better presentation makes it easy to understand.<br>• Thinks about the parent and the child.<br>• Easy to complete.<br>• Captures a good level of detail in covering safety and support (right and left side of the measure respectively)<br>• Some felt the tool was an appropriate length (however, not all agreed)<br>• Co-developed with survivors	• Modifications needed – the tool feels very final.<br>• Not enough focus on feelings but rather the process of safety<br>• Useful for CAFADA services but only works after abuse has been identified.<br>• Leaves out context.<br>• Doesn’t apply to those in the early stages of dealing with abuse.<br>• No reference to online safety

	• Combines relationships (family) and safety in an effective way.<br>• Captures aspects of community – e.g. “me and my family have people who care about us” (but recognized that the exploration of community is dependent on the skills of the practitioner)<br>• Free text box	• Needs a space to define what feel safe means to the responder.<br>• Some questions should be separated so they are easier to answer.<br>• Child measure feels clunky.<br>• Doesn’t consider the parent that harms.<br>• Court-related questions should be reworded as legal aid changes.<br>• Gendered language<br>• Some items are inaccessible to children – does the child have the knowledge to be able to answer this question?<br>• Some issues with inclusivity<br>• Allows only for surface level exploration.<br>• Unsure how to account for the cognitive maturity or neurodivergence

35 Suggested considerations per recommended tool

This table outlines suggested considerations raised during the wider group discussions of the consensus workshop. The considerations documented below include specific recommendations and more general considerations.

	1A: CAFADA Wellbeing and Safety – Relationships Subscale	2A: CAFADA Wellbeing and Safety – Feeling Support Subscale	General considerations
1	Non-traditional family structures are unlikely to access this tool: step-parenting, LGBT parent, extended family, kinship care, foster families, babies and unborn babies, and chosen families.	Online safety is missing given how coercive control can be hidden in use of social media and tracking/surveillance through family media accounts etc.	Consideration is needed for the stage the survivor is in and the possibility of activating feelings of grief/loss – e.g. at the time of abuse, early stages of seeking support, or for families in contact with the court systems
2	The tool uses gendered language and assumes the non-abusive parent is "mum"	The tool doesn't include whether a child continues to have contact with the parent who harms. This is likely to affect safety outcomes	Neurodiversity and cognitive maturity will alter the articulation of feelings – e.g. ASD affecting vulnerability around coercive control but also need/ability to talk about experiences.
3	Phrases such as "difficult times" may be inaccessible and may need more specific examples to support defining these phrases	Cognitive maturity will influence how feelings of safety will be captured as young people's brains are attracted to risk in certain contexts and this should be explored sensitively	Cultures may differ in their comfort levels with sharing certain types of information – further exploration is required to determine whether the instrument behaves differently between cultures/groups.
4	Clarity is needed about the recommended age for children this tool is intended for	While community is important, especially from a faith/cultural perspective, consideration is needed when considering the influence of isolation, mental health, and other intersectional aspects.	Include a "no comment" option for when someone feels unsafe to comment

**5**	Parameters are needed surrounding the free text box, including whether the tool can be used for children to draw their feelings	Framing of community of culture can be interpreted in many different ways – caution against racialization, which can lead to stereotypes/ assumptions/bias; this will be overcome based on the skills of the practitioner.	Presentation of the tool for those with visual impairments
**6**		"Emotional safety" needs further clarification because the term is a broad term and can mean different things for different people	Rigorous translation strategies should be implemented to ensure other versions capture linguistic translations and cultural adaptations.

36 Research team reflexivity statement

The core team working on this study comprised four female researchers, experienced in the field of domestic abuse. The study was co-led by one senior academic with extensive experience researching domestic abuse and one mid-career researcher with experience in domestic abuse research and frontline experience in the mental health sector. The study was project managed by a former independent domestic violence advocate (IDVA) and the data collection was led by a research assistant with experience working with children and families. The team is united by a belief in holding lived experience perspectives central, alongside the views of professionals and researchers. We also believe that academic research needs to be guided by current practice in the voluntary sector (the main service providers in the UK) to help overcome the research-practice gap. The funders, Foundations, have a commitment to equality and diversity and challenged the team to ensure recruitment was focused on minoritised groups.

We wanted to build on our previous work and ensure we included a diverse range of participants. We learned from previous challenges in workshops about how to consider power dynamics; this involved us keeping participant groups separate until the final multi-participant workshop, providing additional support and briefings, following trauma-informed guidelines, and using nominal group technique to ensure everyone had an equal opportunity to speak. Feedback from our lived experience groups suggested we broadly managed to do this although there is always room for improvement.

Our steering group (and co-authors on the paper) were drawn from a range of sectors and services and were a group who were sympathetic to the purpose of the study but also acted as critical friends when needed to highlight gaps and omissions. In particular, they challenged us to think about the broad range of interventions the DA-COS needs to work for and criticisms we might face from the sector.
